# A critical role of an oxygen-responsive gene for aerobic nitrogenase activity in *Azotobacter vinelandii* and its application to *Escherichia coli*

**DOI:** 10.1038/s41598-022-08007-4

**Published:** 2022-03-09

**Authors:** Ren Takimoto, Yuki Tatemichi, Wataru Aoki, Yuishin Kosaka, Hiroyoshi Minakuchi, Mitsuyoshi Ueda, Kouichi Kuroda

**Affiliations:** 1 Division of Applied Life Sciences, Graduate School of Agriculture, Kyoto University, Kitashirakawa Oiwake-cho, Sakyo-ku, Kyoto, 606-8502 Japan; 2 Research and Development Division, Kikkoman Corporation, 338 Noda, Noda, Chiba 278-0037 Japan; 3 Kyoto Monotech, 1095 Shuzei-cho, Kamigyo-ku, Kyoto, 602-8155 Japan

**Keywords:** Microbiology, Molecular biology

## Abstract

Since nitrogenase is irreversibly inactivated within a few minutes after exposure to oxygen, current studies on the heterologous expression of nitrogenase are limited to anaerobic conditions. This study comprehensively identified genes showing oxygen-concentration-dependent expression only under nitrogen-fixing conditions in *Azotobacter vinelandii*, an aerobic diazotroph. Among the identified genes, *nafU*, with an unknown function, was greatly upregulated under aerobic nitrogen-fixing conditions. Through replacement and overexpressing experiments, we suggested that *nafU* is involved in the maintenance of nitrogenase activity under aerobic nitrogenase activity. Furthermore, heterologous expression of *nafU* in nitrogenase-producing *Escherichia coli* increased nitrogenase activity under aerobic conditions by 9.7 times. Further analysis of NafU protein strongly suggested its localization in the inner membrane and raised the possibility that this protein may lower the oxygen concentration inside the cells. These findings provide new insights into the mechanisms for maintaining stable nitrogenase activity under aerobic conditions in *A. vinelandii* and provide a platform to advance the use of nitrogenase under aerobic conditions.

## Introduction

Ammonia is increasingly important in modern society, not only as a fertilizer for plants^1^ but also as a promising molecule to realize a hydrogen society because it is easy to handle^2,3^. Currently, most ammonia is produced by the Haber–Bosch process^4^, which consumes approximately 1–2% of the world’s energy supply^5,6^, and accounts for 1% of the world’s carbon dioxide emissions^7^, making it poorly sustainable. In nature, on the other hand, more than 90% of nitrogen fixation takes place by a process called biological nitrogen fixation (BNF), carried out by diazotrophs^8^.

Diazotrophs are spread among different bacterial phyla and can fix nitrogen at normal temperature and pressure through nitrogenase^9^, a useful property that prompts us to take advantage of them for BNF. For example, several attempts have been made to modify diazotrophs and use them as a substitute for nitrogen fertilizer or to transfer nitrogen-fixing genes to plants^10–16^. However, the complexity of engineering strategies has been a major obstacle in such researches^12,17^. For this reason, there has been a long-standing interest in understanding the genes necessary for nitrogen fixation and reconstituting a nitrogenase biosynthetic pathway by introducing genes from diazotrophs into model organisms^18–21^. Especially in *Escherichia coli*, the minimal gene set required for nitrogenase activity has been clarified^18,19^. Nitrogenase, however, is irreversibly inactivated in a few minutes when exposed to oxygen^22^, and research on the heterologous expression of nitrogenase has been limited to anaerobic conditions.

*Azotobacter vinelandii* can provide useful insights to overcome this limitation. This bacterium is an obligate aerobe that performs nitrogen fixation using nitrogenase, an oxygen-sensitive enzyme. Therefore, there has been an interest in how this bacterium maintains nitrogenase activity under aerobic conditions. The following mechanisms have been proposed to date: physical blockage of oxygen by extracellular alginate production^23^, rapid removal of oxygen by increased respiration rate^24^, and protection of nitrogenase by FeSII protein^25,26^. However, these mechanisms remain inconclusive. For example, alginate production is greatly affected by pH and carbon sources in growth conditions^27,28^. FeSII mutants displayed diazotrophic growth similar to that of the wild-type strain, and FeSII binds only to the molybdenum-iron subunit, not to the vanadium-iron subunit, and it is not clear whether it binds to the iron-only nitrogenase^29^. Furthermore, the protection of nitrogenase by the respiratory system is limited to low dissolved oxygen concentrations, and the consumption of oxygen at the cell surface is not very effective^30,31^. Hence, these three mechanisms alone may not fully explain how *A. vinelandii* maintains high nitrogenase activity under aerobic conditions, suggesting the presence of additional important factors. The identification of such factors would not only advance the study of *A. vinelandii*, but also lead to increased nitrogenase activity in heterologous expression under aerobic conditions since it is possible to introduce additional factors into the heterologous host of nitrogenase^32^.

Transcriptome analysis of *A. vinelandii* has revealed the genes important for ammonia production^33,34^. However, although aerobic physiology is one of the most interesting and important properties of this bacteria, there has been no comprehensive analysis of gene expression in terms of how oxygen affects gene expression. In this study, we conducted a comprehensive search for genes involved in the maintenance of nitrogenase activity under aerobic conditions in *A. vinelandii* focusing on oxygen as well as the presence of nitrogen sources and apply the obtained findings to the heterologous expression of functional nitrogenase under aerobic conditions. With the assumption that the genes necessary for maintaining stable nitrogenase activity under aerobic conditions are expressed in an oxygen concentration-dependent manner under nitrogen-fixing conditions, where a nitrogen source deficiency induces the expression of nitrogenase genes, we performed transcriptome analysis to identify genes showing such an expression pattern. Among the identified genes, we found that *nafU*, whose function is unknown, is strongly induced under aerobic nitrogen fixation conditions and contributes to the maintenance of nitrogen activity under aerobic conditions. Moreover, the additional expression of this gene in nitrogenase-producing *E. coli* increased nitrogenase activity by 9.7-fold under aerobic conditions. Further analysis of this gene revealed that nafU localizes to the inner membrane and is suggested to reduce the intracellular oxygen concentration. The findings of our study will provide new insights into the mechanisms by which *A. vinelandii* stably fixes nitrogen using oxygen-sensitive nitrogenase even under aerobic conditions and a promising strategy for efficient biological use of nitrogenase and other oxygen-sensitive enzymes under aerobic conditions.

## Results

### Identification of genes showing oxygen concentration-dependent expression under nitrogen-fixing conditions

In *A. vinelandii*, the expression of genes involved in maintaining nitrogenase activity under aerobic conditions seems to be induced in an oxygen concentration-dependent manner only under nitrogen-fixing conditions. For example, cytochrome bd is considered to protect nitrogenase from oxygen, and its expression depends on the oxygen concentration under nitrogen-fixing conditions^35,36^. Also, FeSII and the production of alginate, suggested to protect nitrogenase from oxygen, are induced under nitrogen-fixing conditions^29^ and higher oxygen concentrations^23,37^, respectively. These previous results led us to believe that the mechanisms to maintain nitrogenase activity under aerobic conditions are unnecessary and are rather suppressed under low oxygen conditions. Therefore, we assumed that the genes involved in the mechanism are expressed only under nitrogen-fixing conditions, and their expression strongly depends on the oxygen concentration. A comprehensive search for genes showing such an expression pattern could thus discover critical genes involved in protecting nitrogenase from oxygen.

We first examined the culture conditions for identifying these genes by measuring the expression levels of the oxygen-responsive *cydA*, encoding the subunit of cytochrome bd, and *nifH*, encoding the subunit of nitrogenase and being repressed in the presence of nitrogen^33^. The cells pre-cultured in the medium without a nitrogen source were inoculated into the medium with or without a nitrogen source in sealed vials, and the expression level of *nifH* was quantified 2 h and 4 h after the inoculation to determine the optimal incubation time for genes to be induced and suppressed in *A. vinelandii*. As a result, significant difference in expression levels was observed at 2 h compared to 4 h (Fig. S1) to determine the optimal incubation time of 2 h. Next, we examined the oxygen concentrations in the air layer of the vials required for inducing oxygen-responsive genes by quantifying the expression level of *cydA* after 2 h incubation in the medium with or without a nitrogen source under 5%, 10%, or 20% oxygen. As a result, *cydA* expression depended on the oxygen concentration only under nitrogen-fixing conditions (Fig. S2). These results suggest that the incubation of *A. vinelandii* cells under these conditions for 2 h is sufficient to induce and identify a group of genes showing oxygen-concentration-dependent expression only under nitrogen-fixing conditions.

**Figure 1:**
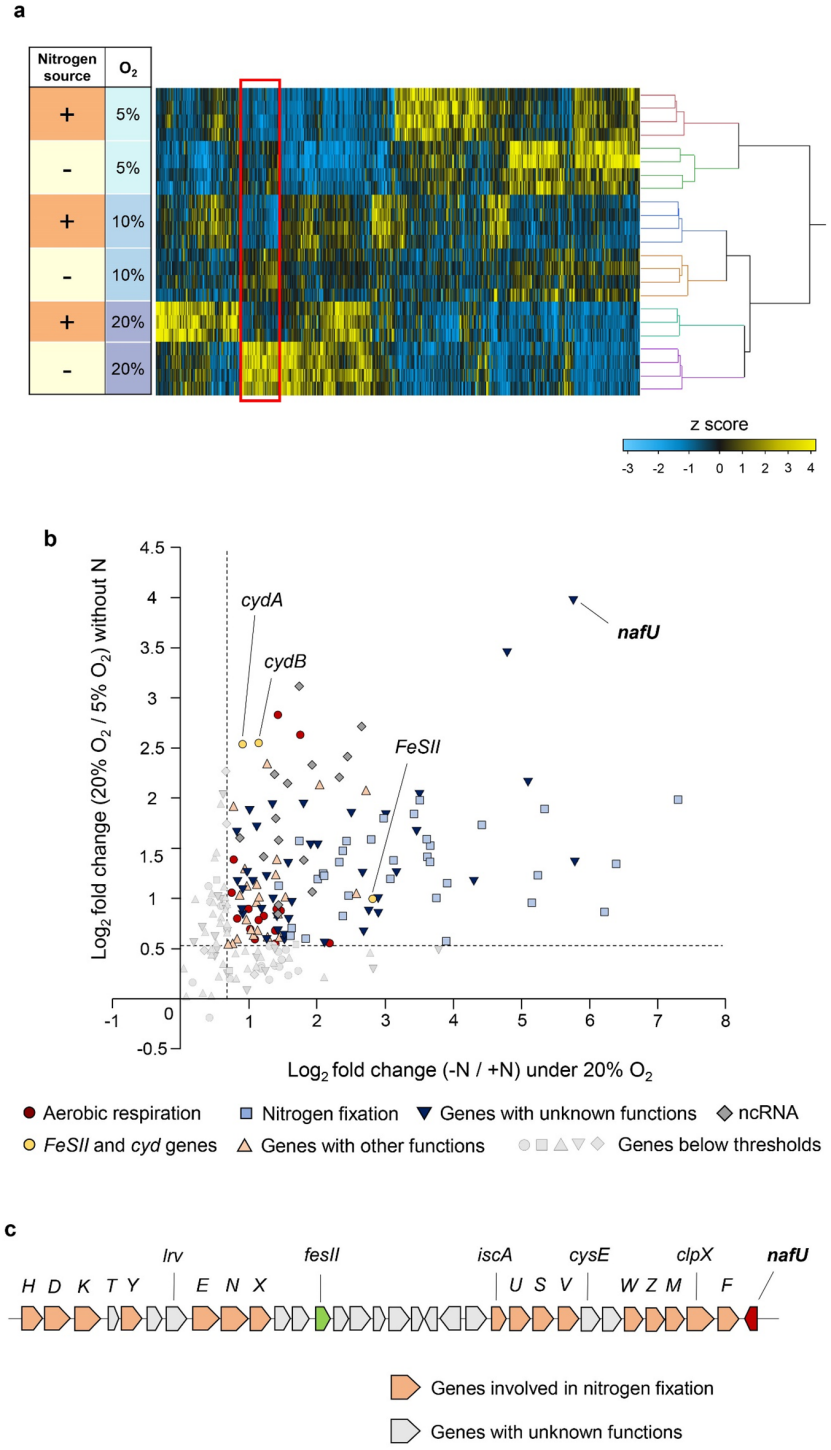
Identification of genes whose expression depends on oxygen concentration only under nitrogen-fixing conditions by transcriptome analysis. **(a)** Heat map generated from the results of transcriptome analysis after ANOVA analysis. The heat map was generated using JMP 16 (https://www.jmp.com). The z-score was calculated for each gene, and genes were clustered using Ward’s method. The cluster surrounded by a red square represents the cluster where genes are upregulated in response to oxygen concentration only without nitrogen source and hardly expressed with nitrogen source. N source “ + ”: with nitrogen source, N source “−”: without nitrogen source, O_2_: O_2_ concentration, n = 3 for under 20% O_2_ with nitrogen source, n = 4 for all other conditions. **b** Fold changes of 213 genes from the cluster in the red square of the heat map **(a)**. Scatter plots show the fold changes of log_2_ (without nitrogen/with nitrogen) under 20% oxygen and log_2_ (20% O_2_/5% O_2_) without nitrogen. Dashed lines indicate cutoffs where log_2_ fold change > 0.58 (fold change > 1.5). Genes below the cut-offs are shown in the shape of each gene classification in gray. (**c)** The chromosomal location of *nafU*. Organization of Mo nitrogenase gene clusters in *A. vinelandii*. The *nif* genes are represented in a single letter.

We conducted transcriptome analysis under the above culture conditions and identified 213 genes whose expressions depended on oxygen concentration only under nitrogen-fixing conditions (Fig. 1a; Table S1). Among these genes, we focused on the 130 genes whose expression was strongly induced under aerobic nitrogen-fixing conditions. Specifically, the focused genes showed increased expression over 1.5-fold in both comparisons: (i) in the medium without a nitrogen source under 20% oxygen compared to under 5% oxygen and (ii) in the medium without a nitrogen source compared to the medium with a nitrogen source under 20% oxygen (Fig. 1b; Table S2). The focused genes included a group of genes with known functions, such as the genes related to aerobic conditions, nitrogen fixation, *FeSII* and *cyd* genes, suggested to contribute to the maintenance of nitrogenase activity under aerobic conditions. Meanwhile, many genes with unknown functions were included. We then focused on *nafU* (Fig. 1c), suggested to be related to ammonia production^34^ but whose function was unknown^38^, because its expression was most induced under aerobic nitrogen-fixing conditions compared with other genes with unknown function (Fig. 1b). This result was validated by RT-qPCR, and we confirmed that *nafU* was strongly induced when *A. vinelandii* was cultured under 20% oxygen without nitrogen (Fig. S3).

### The *nafU* gene maintains nitrogenase activity under aerobic conditions

**Figure 2:**
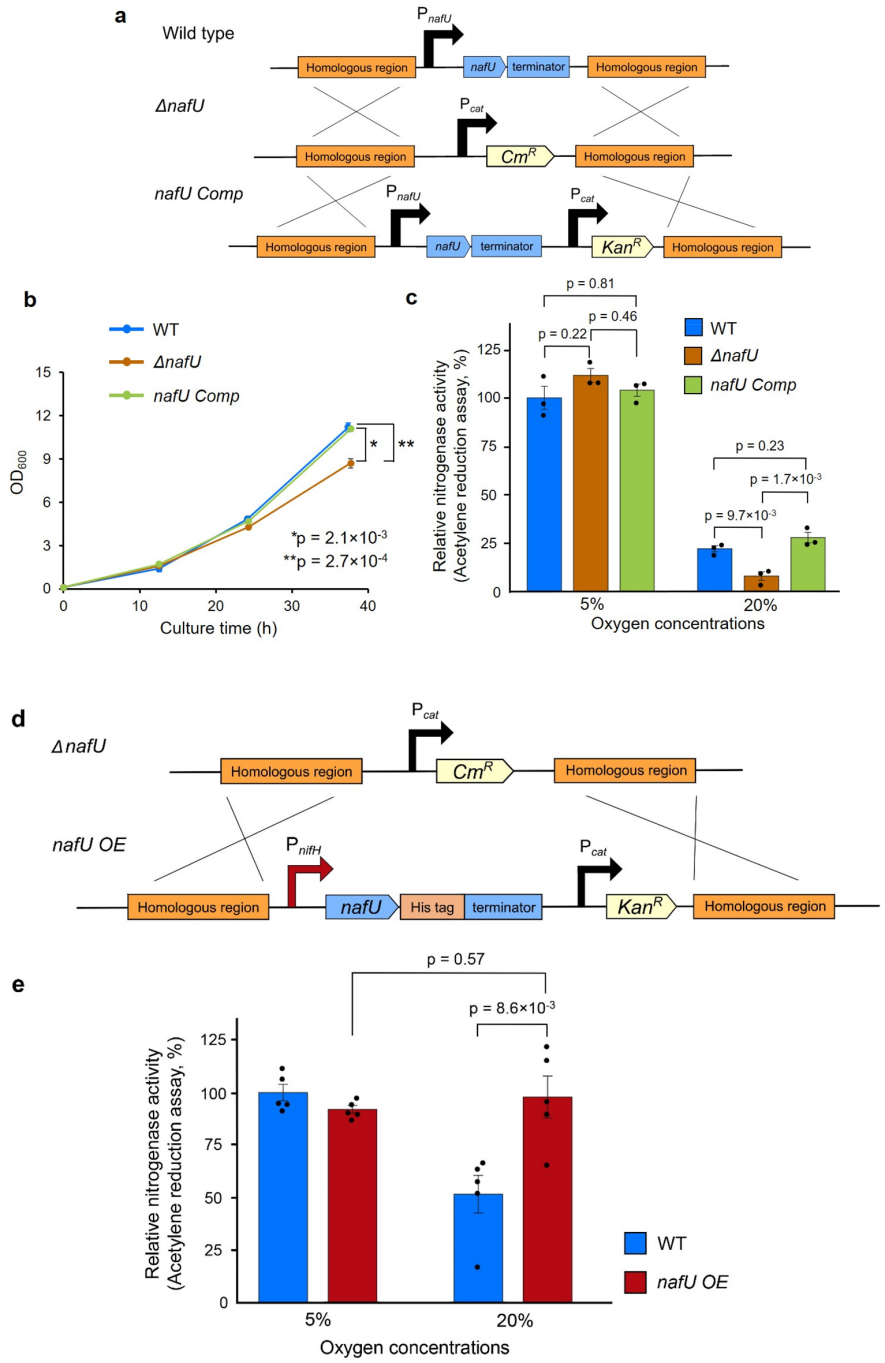
The contribution of *nafU* gene to the maintenance of nitrogenase activity under aerobic conditions. **(a)** Construction of *A. vinelandii ΔnafU* and *nafU comp* strains by genomic insertion. The homologous regions (500–600 bp) upstream and downstream of the *nafU* gene were used for homologous recombination. P_*cat*_, *Cm*^*R*^*,* and *Kan*^*R*^ represent the promoters of chloramphenicol acetyltransferase (cat), chloramphenicol resistant gene, and kanamycin resistant gene, respectively. (**b)** Growth curves of wild-type, *ΔnafU*, and *nafU comp* strains in MB liquid medium without nitrogen source under aerobic conditions. Data are presented as the mean ± SE of three independent experiments. *P-values* were calculated using the Tukey_HSD test. (**c)** Nitrogenase activities of wild-type, *ΔnafU,* and *nafU comp* strains under 5% and 20% oxygen. The cells were sampled at 14 h after the start of culture. The Data are shown as relative values when the average activity in “wild-type strain under 5% oxygen” is 100% (357.49 nmol ethylene h^−1^ mL^−1^ OD_600_^–1^). Data are presented as the mean ± SE of three independent experiments. *P-values* of the results under 5% oxygen and 20% oxygen were calculated using the Tukey_HSD test, respectively. (**d)** Construction of the *nafU OE* strain by genomic insertion. The homologous regions (approximately 1700 bp) upstream and downstream of the *nafU* gene were used for homologous recombination. P_*nifH*_ represents the *nifH* promoter **e** Nitrogenase activity of wild-type and *nafU OE* strains under 5% and 20% oxygen. The cells were sampled at 14 h after the start of culture. Data are shown as relative values when the average activity in “wild-type strain under 5% oxygen” is 100% (479.21 nmol ethylene h^−1^ mL^−1^ OD_600_^–1^). Data are presented as the mean ± SE of five independent experiments. *P-values* were calculated using the Student’s two-sided *t*-test.

To evaluate the effect of *nafU* on nitrogenase activity under aerobic conditions, we constructed the *nafU*-disrupted strain (*ΔnafU*) and *nafU*-complemented strain (*nafU comp*) by genomic insertion (Fig. 2a). RT-qPCR analysis showed that the expression of *nafU* was not detected in the *ΔnafU* strain and was comparable in the *nafU comp* strain to that of WT (Fig. S4). As a result, the final OD_600_ was lower in the *ΔnafU* strain than WT and *nafU comp* in the growth curve in the medium without a nitrogen source under aerobic conditions (Fig. 2b). In addition, we measured the nitrogenase activity of each strain at 5% and 20% oxygen concentrations. In the *ΔnafU* strain, nitrogenase activity was significantly reduced only under 20% oxygen concentration, suggesting that *nafU* contributes in maintaining nitrogenase activity under aerobic conditions (Fig. 2c). To further investigate the positive effect of *nafU* on nitrogenase activity under aerobic conditions, we constructed the *nafU*-overexpressing strain (*nafU OE*) by replacing the promoter of *nafU* with that of *nifH*, strongly induced under nitrogen-fixing conditions (Fig. 2d). Compared with WT, *nafU OE* strain showed about threefold higher transcription and expression of *nafU* (Fig. S5, S12). In addition, the use of the *nifH* promoter for *nafU* overexpression did not significantly reduce the expression of *nifH* in the *nafU OE* strain compared to WT (Fig. S6). Nitrogenase activity of *nafU OE* strain under 20% oxygen concentration was significantly higher than that of WT and showed the same level of nitrogenase activity as that under 5% oxygen (Fig. 2e). These results indicate that *nafU* contributes to the maintenance of nitrogenase activity under aerobic conditions.

### Aerobic nitrogenase activity of nitrogenase-producing *Escherichia coli* is improved by *nafU*

**Figure 3:**
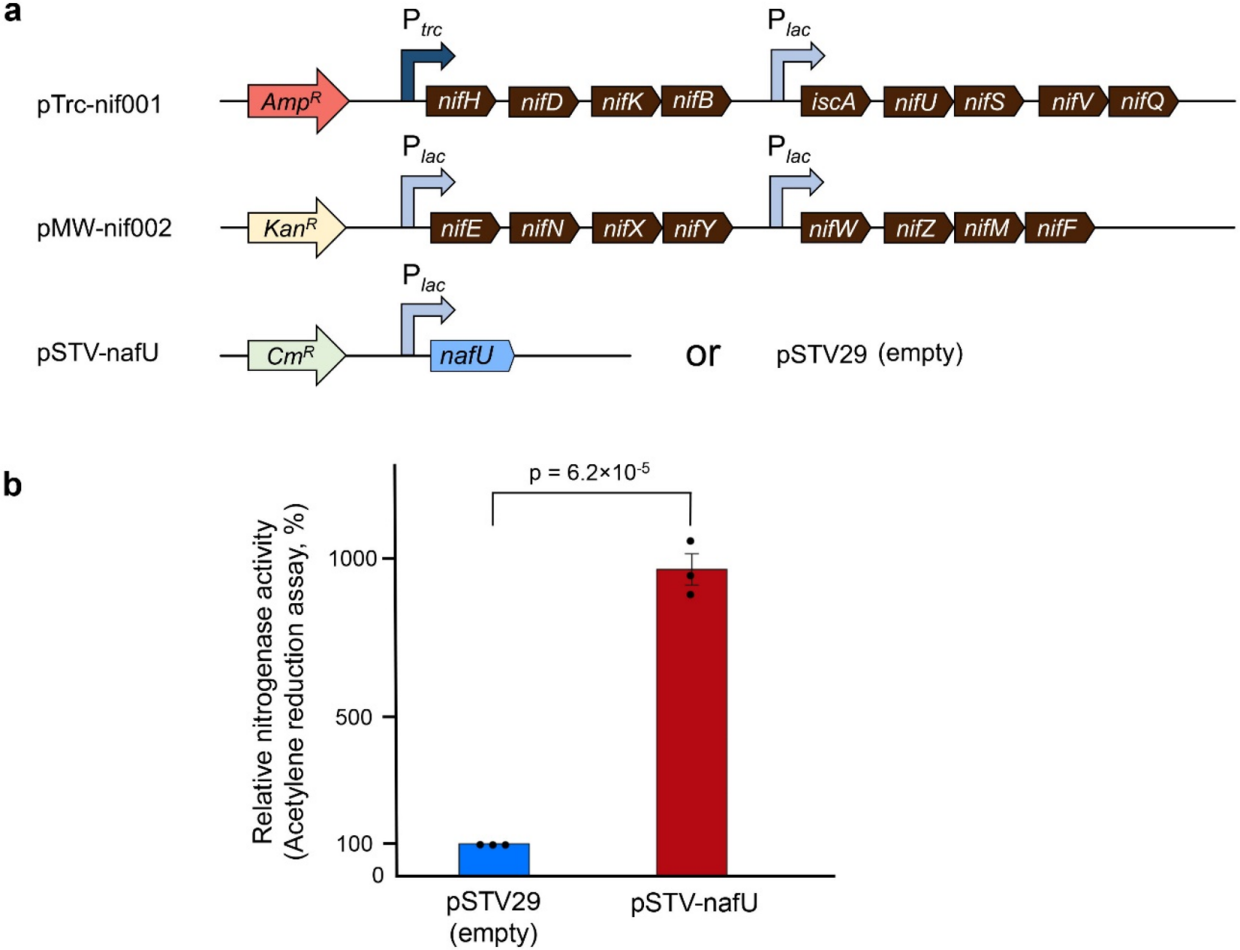
The effect of *nafU* overexpression on aerobic nitrogenase activity in nitrogenase-producing *E. coli.* **(a)** Plasmids for producing nitrogenase and nafU in *E. coli*. Each *nif* gene was cloned from *A. vinelandii* genomic DNA, and *nafU* was codon-optimized for *E. coli*. P_*trc*_ and P_*lac*_ represent the *trc* and *lac* promoters, respectively. pTrc-nif001, pMW-nif002, and pSTV-nafU were constructed from pTrcHis2-TOPO, pMW219, and pSTV29, respectively. (**b)** Nitrogenase activity under 20% oxygen of nitrogenase-producing *E. coli* harboring vector expressing *nafU* or empty vector. Data are shown as relative values when the average activity in *E. coli* harboring “pSTV29 (empty)” is 100% (0.0100 nmol ethylene h^−1^ mL^−1^ OD_600_^–1^). Data are presented as the mean ± SE of three independent experiments. *P-values* were calculated using the Student’s two-sided *t*-test.

*nafU*, identified by transcriptome analysis, was important in maintaining nitrogenase activity under aerobic conditions in *A. vinelandii*. Therefore, the positive impact of *nafU* may apply to the heterologous expression of nitrogenase under aerobic conditions. To test this hypothesis, we utilized the nitrogenase-producing *E. coli* constructed in our previous study^39^. This *E. coli* strain harbors two plasmids with four operons consisting of 17 nif-related genes, cloned from *A. vinelandii* genomic DNA and assigned to each operon based on its expression level. Codon-optimized *nafU* (see “Methods”, Table S6) was overexpressed by introducing the expression vector in nitrogenase-producing *E. coli* (Fig. 3a). As a result, the overexpression of *nafU* greatly increased nitrogenase activity under 20% oxygen by approximately 9.7-fold (Fig. 3b). In contrast, the nafU overexpression did not affect the growth of nitrogenase-producing *E. coli* (Fig. S7). These results indicate that *nafU* is also effective for maintaining nitrogenase activity under aerobic conditions in *E. coli*, suggesting that *nafU* is a useful gene for heterologous expression of nitrogenase under aerobic conditions in other microorganisms.

### Functional estimation of NafU protein

To understand the mechanism by which NafU protein maintains nitrogenase activity under aerobic conditions, we employed an analysis based on the functionally known proteins that are predicted to have a similar structure to NafU protein using a secondary structure prediction tool (JPred4)^40^. As a result, SlyB was predicted to have a secondary structure similar to NafU protein (Fig. S8). In some bacteria, SlyB is localized in the cell membrane and protects the cells from external stresses by increasing the membrane stability^41^. Another protein with a similar function, Hsp12 in yeast, is also known to localize in the membrane and protect the cells from stress by improving the membrane integrity^42^. Based on these analyses, we hypothesized that NafU protein may localize to the cell membrane and protect intracellular nitrogenase from oxygen by improving membrane integrity.

**Figure 4:**
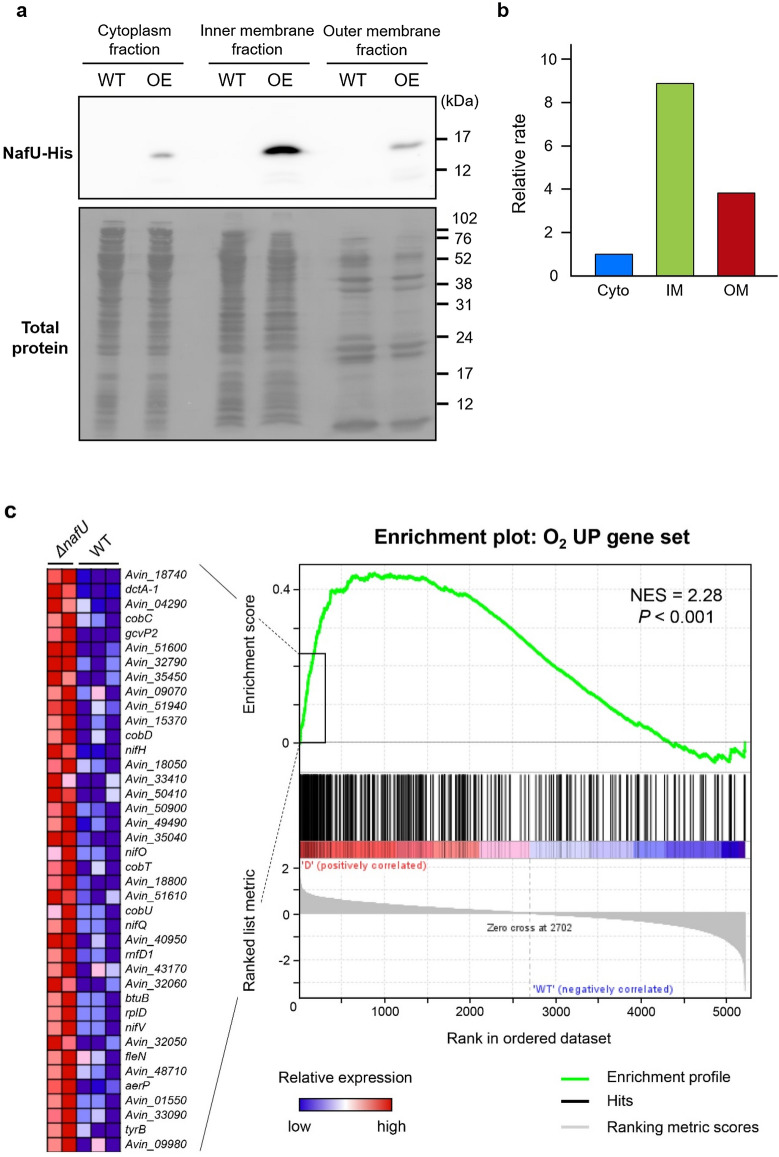

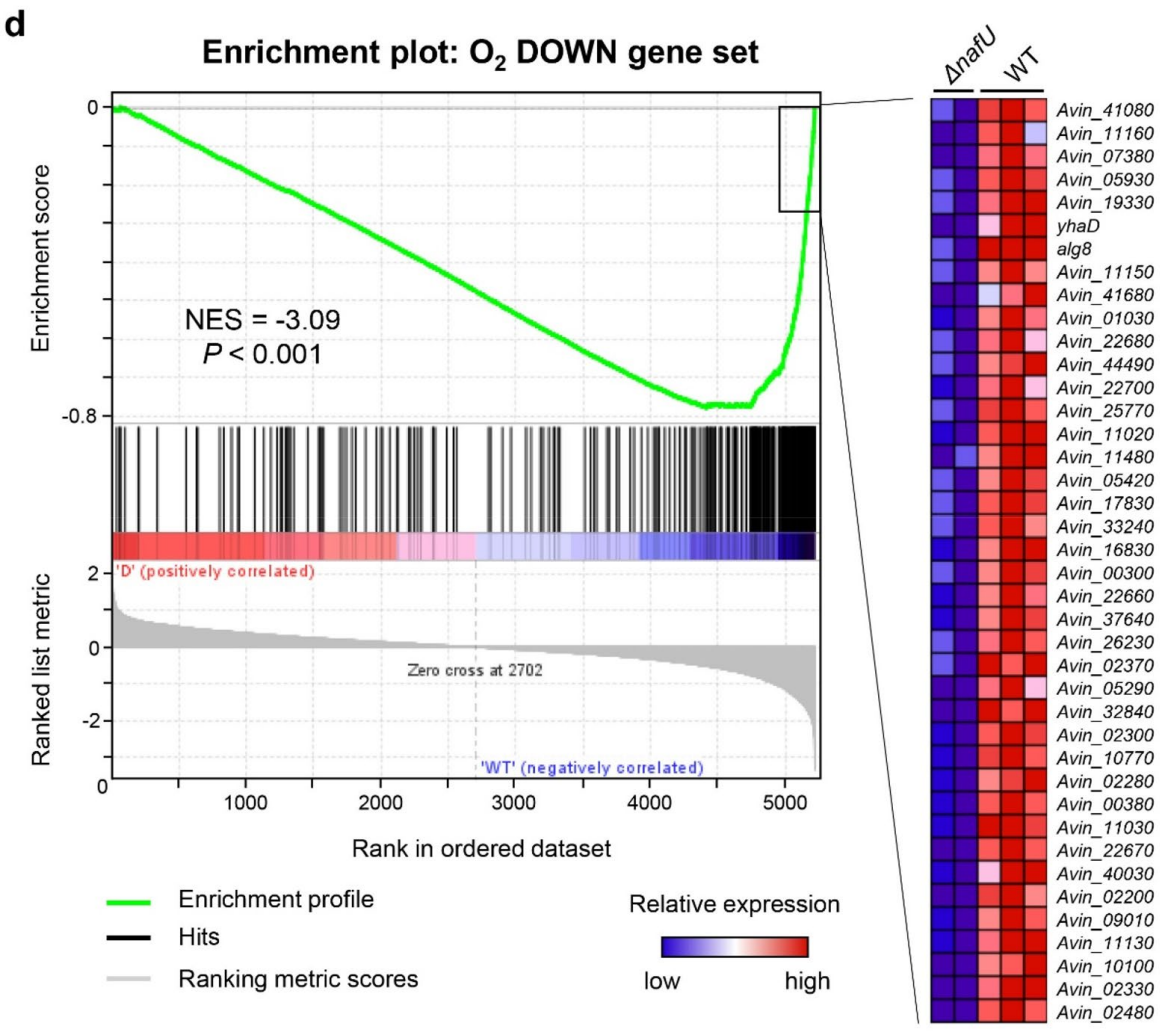
Functional analysis of NafU protein in *A. vinelandii.* **(a)** Cellular localization of NafU examined by western blot analysis of each cellular fraction in wild-type and *nafU OE* strains using an anti-His-tag antibody. NafU-His is 13.8 kDa. A full-length blot is presented in Supplementary Fig. S11. (**b)** Western blot quantification of NafU-His normalized by total protein intensity. *Cyto* cytoplasm, *IM* inner membrane, *OM* outer membrane. Data are shown as relative values when the value of “cytoplasm fraction” is 1. The quantification of the band intensity in western blot analysis and total protein by CBB staining of transferred membrane was performed by ImageJ. (**c,d)** Gene set enrichment analysis (GSEA) of *ΔnafU* versus wild-type strains compared with oxygen-responsive genes. The figures were generated based on the results obtained by using GSEA 4.1.0^47–49^_._ The O_2_ UP gene set included the top 300 genes on log_2_ (20% O_2_/5% O_2_) in the medium without a nitrogen source in transcriptome analysis (Fig. 1a), and the O_2_ DOWN gene set includes the bottom 300 genes. Each gene set is listed in Table S4. The gene groups on the left **(c)** and right **(d)** represent the sets of genes with the largest significant differences in expression levels, and the colors indicate their relative values (red: high, blue: low). (**c)** GSEA summary plot compared to the O_2_ UP gene set. NES (normalized enrichment score) = 2.28 and *P* < 0.001 indicate a positive correlation. (**d)** GSEA summary plot compared with the O_2_ DOWN gene set. NES = −3.09 and *P* < 0.001 indicate a negative correlation.

We first examined the localization of NafU protein by cell fractionation and western blot analysis. The fractionation was validated by a quantitative proteome to examine whether each fraction included proteins with known localization. Proteome analysis showed that soluble cytoplasmic proteins such as NifH^43^ were enriched in the cytoplasmic fraction but not in the membrane fractions. In addition, the proteins that have been shown to localize in the inner membrane, such as CydA^44^, were enriched in the inner membrane fraction, and other outer membrane proteins such as OprI^45^ were enriched in the outer membrane fraction (Fig. S9). Then, each fraction validated by these results was subjected to western blot analysis against the His tag fused to NafU protein. The NafU-His band was detected in the inner membrane fraction (Fig. 4a,b; Fig. S11), indicating that NafU is an inner membrane protein.

Next, we performed transcriptome analysis of WT and *ΔnafU* strains to examine the possibility that NafU protein reduces the intracellular oxygen concentration. The increase or decrease in intracellular oxygen concentration upon *nafU* deletion was examined by the expression changes of oxygen-responsive genes (identified in Fig. 1). Since the transcriptional level of *nafU* was highest around 2 h after inoculation, similar to *nifH* (Fig. S10), WT and *ΔnafU* strains were incubated in the medium without a nitrogen source for 3 h after inoculation to induce the production of NafU protein. After the induction, and the cells were incubated for another 2 h with the vialed being sealed to reproduce the same culture conditions as in the transcriptome analysis in Fig. 1a, followed by transcriptome analysis. Based on the results (Table S3), we performed gene set enrichment analysis (GSEA)^46–48^ for the genes that were induced in response to oxygen (O_2_ UP gene set) and suppressed in response to oxygen (O_2_ DOWN gene set) identified by transcriptome analysis (Fig. 1a) in the medium without a nitrogen source. Specifically, in the transcriptome in the medium without a nitrogen source (Fig. 1a), the top 300 genes on log_2_ (20% O_2_/5% O_2_) were defined as the O_2_ UP gene set and the bottom 300 genes as the O_2_ DOWN gene set (Table S4). The GSEA of the *ΔnafU* strain compared to WT for those gene sets revealed a positive correlation between the genes whose transcription levels were increased in the *ΔnafU* strain and the O_2_ UP gene set, as well as a negative correlation with the O_2_ DOWN gene set (Fig. 4c,d). From these results, there is a possibility that NafU protein functions to decrease intracellular oxygen concentration.

## Discussion

Maintaining the activity of oxygen-sensitive nitrogenase, even under aerobic conditions, has been a challenge for the biological use of nitrogenase via heterologous expression. However, no previous studies have significantly increased nitrogenase activity under aerobic conditions, and the heterologous expression of nitrogenase has so far been conducted only under anaerobic conditions^32,49,50^. Our transcriptome analysis identified the genes that were upregulated in an oxygen concentration-dependent manner under nitrogen-fixing conditions. Among them, we focused on the most upregulated gene, *nafU* with a previously unknown function^38^, and revealed that this gene contributes to the maintenance of nitrogenase activity under aerobic conditions in *A. vinelandii*. We also demonstrated that NafU is an inner membrane protein and suggested the possibility that this protein may reduce intracellular oxygen concentration. Furthermore, by introducing *nafU*, we displayed the improvement of nitrogenase activity of nitrogenase-producing *E. coli* under aerobic conditions, which has not been achieved in previous studies. Therefore, our findings could provide a promising platform for the expression of functional nitrogenase under aerobic conditions in other microorganisms and pave the way for the realization of heterologous aerobic nitrogen fixation.

Our transcriptome analysis also identified genes with known functions such as *nif* genes, involved in nitrogen fixation and expressed in an oxygen concentration-dependent manner under nitrogen-fixing conditions^51^. Therefore, it is conceivable that some genes with unknown functions that show similar expression patterns to *nif* genes could be involved in nitrogen fixation. In addition, many genes involved in aerobic respiration, such as *zwf* ^51^, *ace* ^52^, and *pyk* ^53^ were also part of the identified genes. There are two possible reasons for this finding: (i) cells have to make a lot of energy through respiration for nitrogen fixation, an energy-consuming reaction that requires more than 16 ATP to fix one nitrogen molecule to two ammonia molecules^54^, (ii) *A. vinelandii* prevents oxygen molecules from entering the cells by activating respiration^24^. *Azotobacter vinelandii* has a much faster respiratory rate than other bacteria^55^.

Genes involved in the biosynthesis of alginates, such as *algA* and *algC* ^56^, have also been suggested to maintain nitrogenase activity under aerobic conditions. However, our transcriptome analysis demonstrated that the expression of these genes did not differ significantly between the presence and absence of a nitrogen source in the medium and were rather higher under low oxygen conditions (5%) in terms of RNA levels. This was presumably because *A. vinelandii* is an obligate aerobe and consumed all the oxygen of 5% in the vials by their high respiration, leading to cyst formation that requires alginate biosynthesis^56^. In this regard, the lack of a significant difference in the *nifH* expression level when incubated in sealed vials for 4 h may be due to the induction of cyst formation or cell death by the decrease in oxygen concentration.

A previous study examining gene fitness in *A. vinelandii* by Tn-seq suggested that the deletion of *nafU* does not affect growth under aerobic nitrogen fixing conditions at ~ 7 generations (about 14 h)^57^. This result is consistent with our result at 12 h (Fig. 2b), and the effect on growth can be seen only after increasing incubation time. On the other hand, we found that the deletion of *nafU* significantly reduces nitrogenase activity under aerobic conditions when measured at 14 h after the start of culture (Fig. 2c). Based on our speculation that NafU protein is present in the inner membrane to improve membrane stability, the accumulation of NafU protein in the membrane may be insufficient to be effective and mechanisms other than nafU may be dominant in the early stages of growth.

The *nafU* gene is conserved in the genus *Azotobacter* and some Pseudomonas, according to the results of a BLAST homology search, and does not form an operon with other genes^58^. However, the *nafU* gene is located near the *nif* clusters in the genome (Fig. 1c), suggesting that it has been maintained together as a gene that promotes nitrogen fixation. The upregulation of *nafU* in high-ammonium-excreting *A. vinelandii* strain also supports the importance of this gene in nitrogen fixation^34^. In addition, the secondary structure prediction of NafU protein by JPred4^40^ suggested its similarity in secondary structure to SlyB (Fig. S8), present in some bacteria, localized to the membrane, and increases membrane stability to protect cells from stress^41^. In yeast, there is also a protein with a similar function, Hsp12^42^. In this study, we demonstrated that NafU protein localizes to the inner membrane and suggested that NafU protein may reduce intracellular oxygen concentrations based on the altered transcription of oxygen-responsive genes. These results suggest the possibility that NafU protein can prevent oxygen permeation by increasing inner membrane integrity. Based on this mechanism, it is quite reasonable that the expression of *nafU* was suppressed under low oxygen conditions and the medium with a nitrogen source, because the cells would actively have to take in oxygen and a nitrogen source under such conditions, where the expression of *nafU* is detrimental to survival. To validate this mechanism, further studies are needed on the biochemical aspects of NafU protein, such as its contribution to membrane stability and the identification of important sites for its function.

The *nafU* gene also significantly affected nitrogenase activity positively under aerobic conditions in nitrogenase-producing *E. coli*. However, the nitrogenase activity of nitrogenase-producing *E. coli* with *nafU* was still low compared to that of *A. vinelandii*. Since genes involved in aerobic respiration were up-regulated under aerobic nitrogen-fixing conditions, these genes may also contribute to maintaining nitrogenase activity under aerobic conditions. Thus, the additional expression of these genes and *nafU* in the nitrogenase-producing *E. coli* may further increase the activity.

In conclusion, this study provides new insights into the mechanism by which *A. vinelandii* maintains a stable nitrogenase activity even under aerobic conditions. Furthermore, our findings can be applied to the heterologous expression of nitrogenase in *E. coli* to increase nitrogenase activity under aerobic conditions. This improvement has not been achieved in previous studies. In addition to nitrogenase, other oxygen-sensitive enzymes, such as hydrogenase, are useful but have not been used effectively because of their oxygen sensitivity. Therefore, our findings would greatly contribute to realizing the heterologous expression of active nitrogenase under aerobic conditions and the stable heterologous expression of other oxygen-sensitive enzymes under aerobic conditions.

## Methods

### Strains, media, and culture conditions

*Azotobacter vinelandii* Lipman (ATCC 9046) and its derivatives used in this study are listed in Table S5. The *A. vinelandii* strains were cultured on a modified Burk’s medium plate^59^ (0.66 g/L K_2_HPO_4_, 0.16 g/L KH_2_PO_4_, 0.2 g/L NaCl, 2.9 mg/L Na_2_MoO_4_·2H_2_O, 20 g/L sucrose, 0.04 g/L CaCl_2_·2H_2_O, 0.2 g/L MgSO_4_·7H_2_O, 27 mg/L FeSO_4_·7H_2_O, and 15 g/L agar) at 30 °C, where 1.5 g/L NH_4_(CH_3_COO) was added when cultured with nitrogen. MB^+^ and MB^-^ represent the modified Burk’s medium with and without nitrogen, respectively. For the culture in a liquid medium, agar was not added, and cells were incubated at 30 °C and 300 rpm if not specified.

*Escherichia coli* strains DH5α (Takara Bio, Shiga, Japan) and JM109 (TaKaRa Bio) used in this study are listed in Table S5. The DH5α strain was used as the host for recombinant DNA manipulation. DH5α transformant cells were grown in LBA medium (1% [w/v] tryptone, 0.5% [w/v] yeast extract, and 0.5% [w/v] sodium chloride) containing 100 mg/L ampicillin. For the acetylene reduction assay on *E. coli*, the JM109 strain was used as a host for nitrogenase production. For measurement of the nitrogenase activity, KPM minimal medium (4 g/L Na_2_HPO_4_, 3.4 g/L KH_2_PO_4_, 26 mg/L CaCl_2_·2H_2_O, 30 mg/L MgSO_4_, 0.3 mg/L MnSO_4_, 41 mg/L FeSO_4_·7H_2_O, 10 mg/L para-aminobenzoic acid, 5 μg/L biotin, 337 mg/L vitamin B1, 4 g/L glucose, and 7.6 mg/L Na_2_MoO_4_) containing antibiotics (100 mg/L ampicillin, 25 mg/L kanamycin, and 10 mg/L chloramphenicol) was used.

### RNA isolation

For transcriptome analysis, *A. vinelandii* strains were cultured on MB^+^ plates for 2 days, inoculated into MB^-^ liquid medium with an initial OD_600_ of 0.1, and incubated for 24 h. Next, the cells were harvested, washed with ice-cold phosphate solution (0.1 g K_2_HPO_4_ and (0.4 g KH_2_PO_4_ in 450 mL distilled water), and inoculated into 25 mL of MB^+^ or MB^-^ liquid medium with an initial OD_600_ of 0.5. The culture vials were purged with N_2_ for 1 min and sealed, and then O_2_ was injected into the air layer to bring the O_2_ concentration to 5%, 10%, or 20%. After 2 h of incubation, an equal volume of ice-cold phosphate solution was added to the culture medium, and the cells were collected at 3000×*g* for 2 min. After removing the supernatant, the cell pellets were flash-frozen in liquid nitrogen and stored at −80 °C until needed. When measuring the expression levels of *nafU* (Figs. S4 and S5), the cells were cultured on MB^+^ plates for 2 days, in MB^+^ liquid medium for 24 h, and in MB^-^ liquid medium for 2 h. In the transcriptome analysis of WT and *ΔnafU* strains (Fig. 4c,d), the cells were cultured on MB^+^ plates for 2 days, in MB^+^ liquid medium for 24 h, and in MB^-^ liquid medium for 3 h. Then, the vials were sealed, and the cells were collected after an additional 2 h of incubation.

Total RNA was extracted using TRIzol reagent (Invitrogen, NY, USA) according to the manufacturer’s instructions. RNase-free DNase I (NIPPON GENE, Tokyo, Japan) were used with RNase Inhibitor (TOYOBO, Osaka, Japan) to remove the residual genomic DNA. The quantity and quality of the isolated RNA were measured using a NanoDrop 2000 spectrophotometer (Thermo Fisher Scientific, MA, USA) and BioAnalyzer 2100 (Agilent Technologies, CA, USA), respectively. For RNA-seq analysis, rRNAs were removed using the RiboMinus Transcriptome Isolation Kit (Thermo Fisher Scientific). The concentration of mRNA was measured by Bioanalyzer 2100 using an RNA 6000 Nano Kit (Agilent Technologies).

### Reverse transcription-quantitative PCR (RT-qPCR) analysis

cDNA was synthesized from the isolated RNA using a High-Capacity cDNA Reverse Transcription Kit (Thermo Fisher Scientific) according to the manufacturer’s instructions. Quantitative real-time PCR (qPCR) was performed using Fast Start SYBR Green Master (Roche, Mannheim, Germany) or Power SYBR Green PCR Master Mix (Thermo Fisher Scientific), and the measurement was performed using a C1000 Touch Thermal Cycler (Bio-Rad, CA, USA). The sequences of the primers used for qPCR are listed in Table S6. The expression level of *gyrA* was used as an internal control^60^ to normalize that of *nifH* and *cydA* (Figs. S1 and S2), and the expression level of *rho* was used for the other experiments (Figs. S3, S4, S5a, S6, and S10) because the transcriptome analysis results (Fig. 1a) showed that the expression level of *rho* had less variation among the culture conditions. The relative gene expression was quantified using the standard curve method.

### RNA-seq analysis

Libraries were prepared using the KAPA RNA HyperPrep Kit (Kapa Biosystems, MA, USA). The prepared cDNA libraries were validated with BioAnalyzer 2100 using a High Sensitivity DNA Kit (Agilent Technologies Inc.) and sequenced on an Illumina MiSeq (v3 reagent, 75-bp nucleotide paired-end sequence) to search for candidate genes (Fig. 1a) and HiSeq X Ten (150 bp nucleotide paired-end sequence) to estimate the function of *nafU* (Fig. 4b,c). All raw data files were deposited in the ArrayExpress.

The RNA-seq reads were aligned to the *A. vinelandii* DJ (CP001157.1) genome sequence using EDGE-Pro^61^. The read counts were extracted and normalized using edgeR^62^. The heat map was created using JMP 16 (SAS Institute Inc., Cary, NC, USA) for genes that showed significant differences by ANOVA analysis.

GSEA analysis using GSEA 4.1.0^46–48^ was performed on the transcriptome data after alignment and normalization to determine the function of NafU protein. The transcriptomic results of WT and *ΔnafU* strains were subjected to enrichment analysis in the absence of nitrogen (Fig. 1a) to examine the effect of NafU protein on intracellular oxygen levels. Specifically, among the genes included in the cluster diagram shown in Fig. 1a, in the medium without a nitrogen source, the top 300 genes on log_2_ (20% O_2_/5% O_2_) were defined as the O_2_ UP gene set and the bottom 300 genes as the O_2_ DOWN gene set (Table S4). GSEA analysis was performed with default settings for these gene sets, except phenotype labels being set as *ΔnafU* vs. WT, Collapse/Remap to gene symbols as No_Collapse, and Permutation type as a gene set.

### Construction of *A. vinelandii* strains

All primers used for the construction of the *A. vinelandii* strains are listed in Table S6. DNA fragments were combined with linearized vectors by In-Fusion HD Cloning Kit (Takara Bio). For the construction of the strain with a disruption of *nafU* (*ΔnafU*), homologous sequences of 500–600 bp in the downstream and upstream of *nafU* gene were amplified by PCR from the *A. vinelandii* genomic DNA extracted with the DNeasy Blood & Tissue Kits (QIAGEN, Hilden, Germany) using primer sets of “*nafU DIS* A” and “*nafU DIS* B” , respectively. Then, these fragments were combined with linearized pUC19 (Thermo Fisher Scientific) by PCR using a primer set “pUC19,” flanking the chloramphenicol resistance gene amplified from pSTV29 (Takara Bio) using a primer set of “Chloramphenicol.”. The constructed plasmid was named pUC19-*ΔnafU* and introduced into *A. vinelandii* to delete *nafU* by genome insertion, according to previous reports^54,63^. The *ΔnafU* cells were selected on an MB^+^ plate containing 170 mg/L chloramphenicol.

The *nafU* gene was complemented in the *ΔnafU* strain as below. The DNA fragment containing 647 bp downstream of *nafU* and the fragment containing 577 bp upstream of *nafU*, *nafU* ORF, and its terminator were amplified using “*nafU comp* A” and “*nafU comp* B,” respectively. The fragment of the kanamycin resistance gene amplified from pMW219 (NIPPON GENE) using a primer set of “Kanamycin” was connected downstream of *Cat* promoter amplified by a primer set of “*Cat* promoter.” These fragments were combined with linearized pUC19. The constructed plasmid was named pUC19-*nafU comp* and introduced into the *ΔnafU* strain by genome insertion. The *nafU Comp* strain was selected on an MB^+^ plate containing 2 mg/L kanamycin.

The strain overexpressing *nafU* was constructed as follows. Homologous sequences of approximately 1700 bp downstream and upstream of the *nafU* gene were amplified from the genomic DNA of *A. vinelandii* using primer sets “*nafU OE* A” and “*nafU OE* B”, respectively. These fragments were combined with linearized pUC19, the fragment consisting of the Kan resistant gene under *Cat* promoter, the terminator of *nafU*, and a part of His tag amplified from pUC19-*nafU comp* using a primer set of “Kan + P_*cat*_ + terminator + His”, the fragment containing *nafU* and a part of His tag amplified from the genomic DNA of *A. vinelandii* using a primer set of “His + nafU”, and the fragment of *nifH* promoter amplified from the genomic DNA of *A. vinelandii* using a primer set of “*nifH* promoter”. The constructed plasmid was named pUC19-*nafU OE* and introduced into the *ΔnafU* strain. The *nafU OE* strain was selected in the same way as the *nafU comp* strain.

The *nafU-His* strain under the native promoter was constructed using almost the same method as the *nafU OE* strain with an upstream homologous region designed to contain the *nafU* gene. The primer sets “*nafU* Native A, “*nafU* Native B”, and “Kan + P_*cat*_ + terminator + His” were used. The constructed plasmid was named pUC19-*nafU-His* under the native promoter.

The construction of the above *A. vinelandii* strains was confirmed by Sanger sequencing of the inserted DNA region.

### Cell fractionation

Cell fractionation was performed using a previously described protocol^64^ with some modifications. *A. vinelandii* WT and *nafU OE* strains were cultured on MB^+^ plates for 2 days and inoculated into MB^+^ liquid medium with an initial OD_600_ of 0.1. After cultivation for 24 h, the cells were collected, washed with ice-cold phosphate solution, and incubated in MB^-^ liquid medium for 4 h with an initial OD_600_ of 0.2. Cell pellets were washed three times with PBS (pH 7.4) containing 15 mM EDTA to reduce cell aggregation and were frozen in liquid nitrogen. The frozen pellets were suspended in ice-cold 10 mM Tris–HCl (pH 8.0) and sonicated on ice using a sonicator (Q125, QSONICA, CT, USA) at 20 kHz and 50% amplitude for 10 s pulse with 10 s intervals until the cumulative J reached 300 J. After sonication, unbroken cells and debris were pelleted at 18,000×*g* for 5 min at 4 °C. The supernatant was collected, and the pellet was resuspended in ice-cold 10 mM Tris–HCl (pH 8.0), followed by sonication under the same conditions as above. This procedure was repeated four times to completely disrupt the cells. The lysate was then ultracentrifuged at 100,000×*g* for 90 min at 4 °C, and the supernatant was collected as the cytoplasmic fraction. The pellet was suspended in 12 mL of ice-cold 10 mM Tris–HCl (pH 8.0) containing 1.7% sarkosyl on ice and incubated for 20 min on ice. The supernatant after ultracentrifugation at 100,000×*g* for 90 min at 4 °C was collected as the inner membrane fraction. The pellet was suspended in 12 mL of PBS containing 2% (w/v) SDS and incubated at 95 °C for 30 min. After centrifugation at 5700×*g* to remove cellular debris, the supernatant was collected as the outer membrane fraction. Each fraction was concentrated by ultrafiltration using an Amicon Ultra-15 centrifugal filter unit (Merck, Darmstadt, Germany) according to the manufacturer’s instructions.

To confirm the overexpression of *nafU* in the constructed strain *nafU OE* (Fig. S5, S12), only the membrane protein was extracted as follows. The *nafU-His* strain that produces nafU-His under the control of the native promoter and *nafU OE* strain were cultured under the same conditions as above. The harvested cell pellets were frozen with liquid nitrogen, suspended in 100 μL of PBS containing 15 mM EDTA per 0.1 g of wet pellet weight, and sonicated as above. The lysate was then ultracentrifuged at 100,000×*g* for 90 min at 4 °C, and the pellet was suspended in PBS containing 2% (w/v) SDS and boiled at 95 °C for 30 min. The solution was then centrifuged at 5700×*g* for 5 min at RT, and the supernatant was collected as the membrane fraction. Each fraction was concentrated by ultrafiltration using an Amicon Ultra-15 centrifugal filter unit (Merck, Darmstadt, Germany) according to the manufacturer’s instructions.

### Western blot analysis

Each protein sample was separated by 12% (w/v) SDS-PAGE and transferred onto a polyvinylidene fluoride (PVDF) membrane. The membrane was blocked with 5% skim milk in PBS, probed with anti-His-tag mAb-HRP-DirecT (MBL, Tokyo, Japan) at a 1:5000 dilution, and visualized using Chemi-Lumi One Super (Nacalai Tesque, Kyoto, Japan).

The protein-transferred PVDF membrane was stained with 0.025% (w/v) CBB in 40% (v/v) MeOH for total protein normalization, destained with 50% (v/v) MeOH containing 7% (v/v) acetic acid, and scanned for ImageJ quantification to confirm the overexpression of *nafU* at the protein level. After scanning, complete decolorization was carried out using 50% MeOH containing 25% (v/v) acetic acid, followed by western blot analysis using an anti-His-tag mAb-HRP-DirecT.Blocking was performed with 0.3% (w/v) skim milk in TBS containing 0.05% (v/v) Tween 20, and the antibody was diluted with IMMUNO SHOT (COSMO BIO, Tokyo, Japan) to increase the band intensity.

### Preparation of protein digests

The protein sample of each fraction was subjected to chloroform/methanol precipitation^65^ and the pellet was dissolved in solubilization buffer (4.97 mg sodium deoxycholate and 3.74 mg sodium N-lauroylsarcosinate in 1 mL of 50 mM TEAB solution). The protein concentration was determined by BCA assay. The samples were reduced and alkylated by incubation with 50 mM DTT at 37 °C for 15 min and 50 mM IAA at RT for 30 min. Next, the samples were mixed with Trypsin/Lys-C Mix (Promega, WI, USA) at a total protein: enzyme volume ratio of 50:1, and digested by shaking at 37 °C for 24 h and the reaction was stopped by 0.5% (v/v) trifluoroacetic acid. The digestion was confirmed by 12% SDS-PAGE with silver staining (Sil-Best Stain One, Nacalai Tesque). The digested peptides were desalted using a MonoSpin C18 column (GL Sciences, Tokyo, Japan). The desalted samples were labeled with TMTsixplex™ Isobaric Label Reagent (Thermo Fisher Scientific) according to the manufacturer’s instructions, and 60 μL of each labeled sample was mixed. The mixture was dried by vacuum centrifugation for the proteome analysis.

### Proteome analysis

Proteome analysis was performed in the previously optimized method using an LC–MS system (LC, UltiMate 3000 RSLCnano System, and MS, LTQ Orbitrap Velos mass spectrometer; Thermo Fisher Scientific) equipped with a long monolithic silica capillary column (490 cm in length, 0.075 mm ID; Kyoto Monotech, Kyoto, Japan)^66,67^. All raw data files were deposited in the jPOST database^68^. We performed the data analysis according to the previously described method^69^ with an exception of using the NCBI protein database of *A. vinelandii* DJ (CP001157.1) for alignment.

### Measurement of acetylene reduction by *A. vinelandii*

After cultivating each strain on MB^+^ plates for 2 days, the cells were collected, washed with phosphate solution, and inoculated into MB^+^ liquid medium with an initial OD_600_ of 0.1. After 24 h of incubation, an equal volume of ice-cold phosphate solution was added to the culture, and the cells were collected at 5700×*g* for 5 min at 4 °C and washed three times with phosphate solution. The cells were then incubated in MB^-^ liquid medium with an initial OD_600_ of 0.1, at 30 °C and 250 rpm for 14 h under aerobic conditions. After incubation, 2.5 mL of the cell culture and 2.5 mL of MB^-^ liquid medium were placed into a 17 mL vial and flashed with Ar for 1 min, followed by sealing with a butyl rubber (GL Science). In the vials, 5% or 20% of the headspace gas was replaced with O_2_, and 10% of the headspace gas was replaced with acetylene generated from CaC_2_. After incubation for 60 min with shaking at 30 °C and 250 rpm, the reaction was stopped by soaking the vials in ice water. Next, the headspace gas (100 μL) was injected into a GCMS-QP2010 (Shimazu, Kyoto, Japan) connected to an HP-PLOT Q column (30 m × 0.32 mm diameter with 20 μm film), and the amount of ethylene was quantified based on ethylene of known concentration. The injector, detector, and oven temperatures were 50 °C, 200 °C, and 35 °C, respectively. The amount of produced ethylene was normalized by OD_600_ value of each sample.

### Construction of nitrogenase-producing *E. coli* expressing *nafU*

Nitrogenase-producing *E. coli* was constructed using previously described methods^39^. Briefly, 17 nif-related genes (*nifH, nifD, nifK, nifB, iscA, nifU, nifS, nifV, nifQ, nifE, nifN, nifX, nifY, nifW, nifZ, nifM,*and *nifF*) were cloned from the genomic DNA of *A. vinelandii*. The PCR amplicons of these genes were assembled by overlap extension PCR using the gene cluster (*nifH*, *nifD*, *nifK*, and *nifB*) under the *trc* promoter and the gene cluster (*iscA*, *nifU*, *nifS*, *nifV*, and *nifQ*) under the *lac* promoter, and fused to PCR-amplified pTrcHis2-TOPO (Thermo Fisher Scientific) using the In-Fusion HD Cloning Kit. Likewise, the gene cluster (*nifE, nifN, nifX,* and *nifY*) and the gene cluster (*nifW, nifZ, nifM,* and *nifF*) were assembled under the *lac* promoter from each PCR amplicon and fused to PCR-amplified pMW219. The resulting plasmids were named pTrc-nif001 and pMW-nif002, respectively. The *nafU* gene codon-optimized for *E. coli* (Table S6) was artificially synthesized (Integrated DNA Technologies, IA, USA). The gene fragment and pSTV29 linearized by PCR were assembled using the In-Fusion HD Cloning Kit to obtain pSTV-nafU. pSTV-nafU or pSTV29 was transformed into nitrogenase-producing *E. coli* JM109 harboring two plasmids, pTrc-nif001 and pMW-nif002 expressing *nif* related genes.

### Measurement of acetylene reduction by nitrogenase-producing *E. coli*

Nitrogenase activity of recombinant *E. coli* was performed as previously described^19^. Briefly, each strain was cultured in LB medium at 37 °C and 200 rpm overnight, and the cells were collected by centrifugation at 3000×*g* for 5 min at 25 °C. The cell pellet was inoculated in 5 mL of KPM medium containing 0.1 mM IPTG and antibiotics with an initial OD_600_ of 0.5, in a 21 mL headspace vial. The headspace was replaced with Ar multiple times and sealed with a butyl rubber septum. Then, 1 mL of a gas mixture of 1:200 ethane gas and acetylene gas obtained from CaC_2_ was added to the vial using a syringe. After incubation at 30 °C for 16 h, the reaction was stopped by injecting 300 µL of 4 M NaOH into each vial. The produced ethylene (*m/z*: 26) and the internal standard ethane (*m/z*: 30) were detected using GCMS QP2020Ultra (Shimadzu) connected to a TurboMatrix HS110 headspace autosampler (PerkinElmer, MA, USA) and GS GasPro column (0.32 mm, 30 m) (Agilent Technologies), and the concentration was determined by the external standard method. The injector, detector, and oven temperatures were 40 °C, 250 °C, and 40 °C, respectively. The average of the results in samples without *E. coli* inoculation was used as the negative control and was subtracted from the average values in samples with *E. coli*.

## Supplementary Information

Supplementary Figures.

Supplementary Tables S1 - S4 .

Supplementary Tables S5 - S6.

## Data Availability

All data and materials that support the findings of this study are available from the corresponding author upon reasonable request. The raw data of the transcriptome analysis were deposited in ArrayExpress: E-MTAB-10710 for MiSeq data and E-MTAB-10712 for HiSeq X. The raw data of the proteome analysis were deposited in the jPOST database: JPST001241 and PXD027133.
