# Supplementary material for: A critical role of an oxygen-responsive gene for aerobic nitrogenase activity in Azotobacter vinelandii and its application to Escherichia coli
Source: Sci Rep. 2022 Mar 9;12:4182. doi: 10.1038/s41598-022-08007-4 (PMC8907163; doi:10.1038/s41598-022-08007-4)
Supplement: Supplementary file 1 — Supplementary Figures. [file 41598_2022_8007_MOESM1_ESM.pdf]

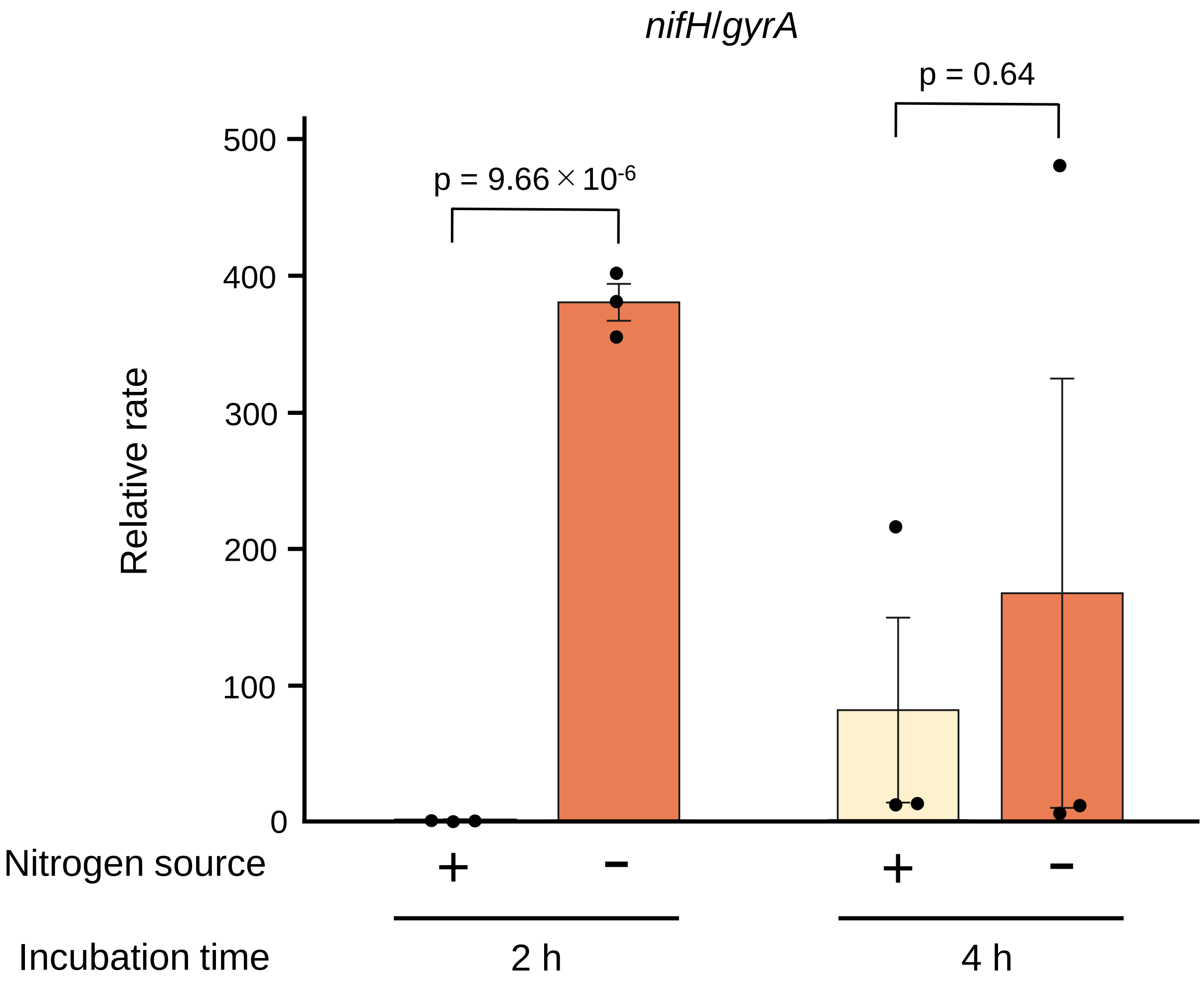

Supplementary Figure 1. The expression levels of *nifH* 2 h and 4 h after incubation in MB liquid medium with or without a nitrogen source. The expression levels were quantified by RT-qPCR, and the expression level of *gyrA* was used for normalization. Data are shown as relative values when the average expression level of the sample “with nitrogen at 2 h” is 1. Data are presented as the mean ± SE of three independent experiments. *P-values* were calculated using the Student’s two-sided t-test.

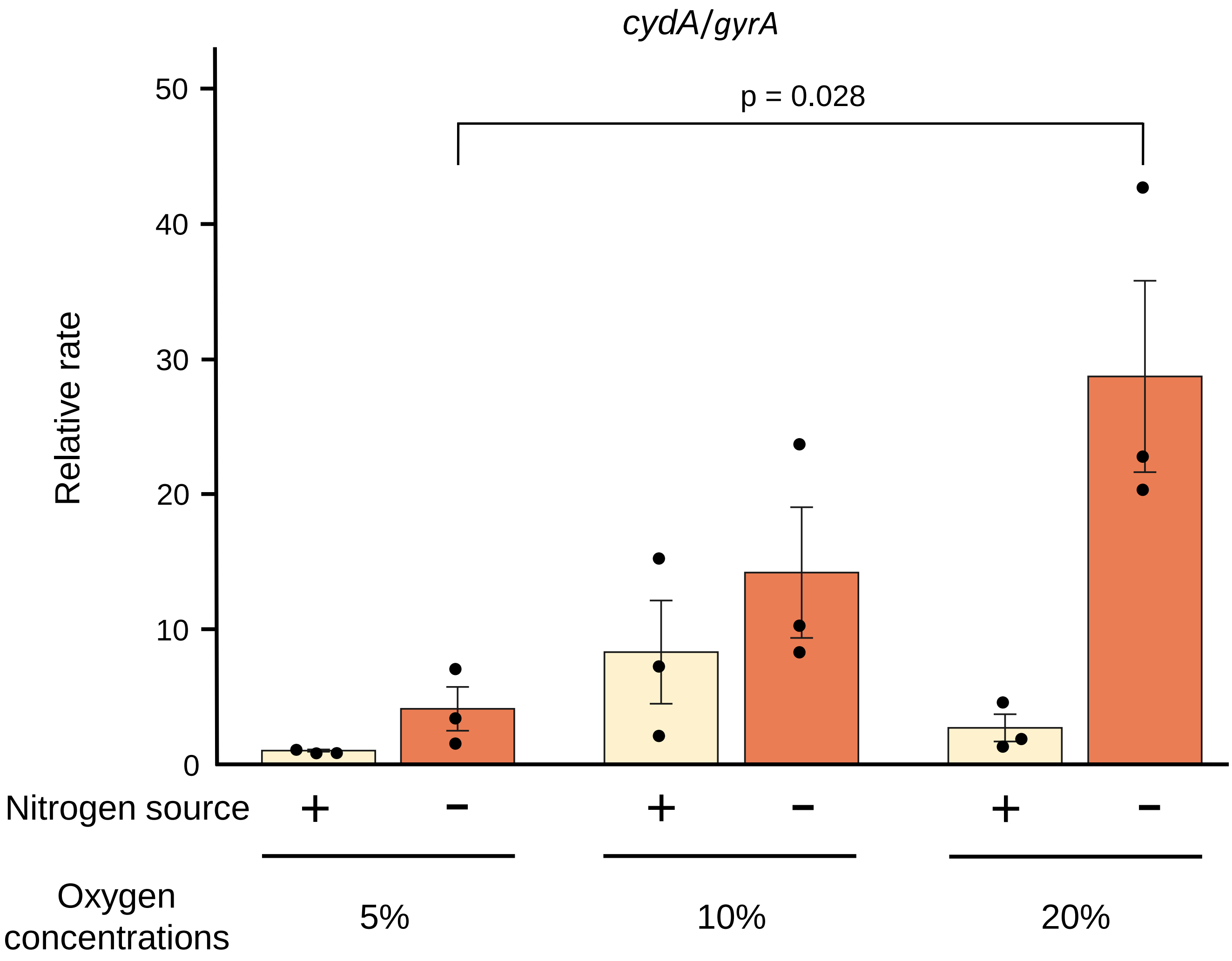

Supplementary Figure 2. The expression levels of *cydA* under 5%, 10%, and 20% oxygen 2 h after incubation in MB liquid medium with or without a nitrogen source. The expression levels were quantified by RT-qPCR and the expression level of *gyrA* was used for normalization. Data are shown as relative values when the average expression level of the sample under “5% O_2 with nitrogen” is 1. Data are presented as the mean ± SE of three independent experiments. *P-values* were calculated using the Student’s two-sided t-test.

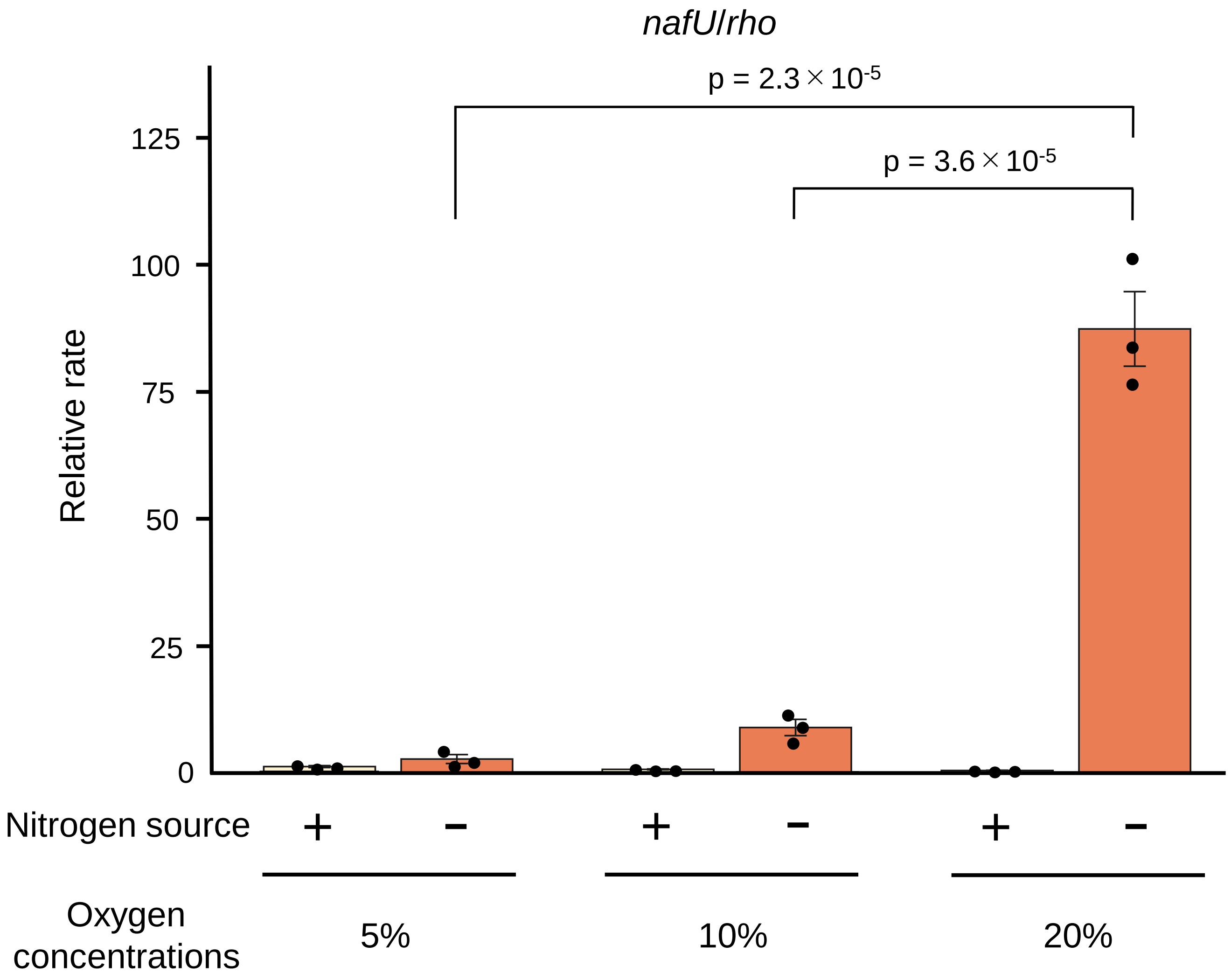

Supplementary Figure 3. Validation of the RNA-seq result of *nafU* expression level by RT-qPCR. *nafU* is strongly induced when the cells are cultured under 20% oxygen without a nitrogen source. The expression level of *rho* was used for normalization. Data are shown as relative values when the average expression level of the sample under "5% O_2 with nitrogen" is 1. Data are presented as the mean ± SE of three independent experiments. *P-values* were calculated using the Tukey_HSD test.

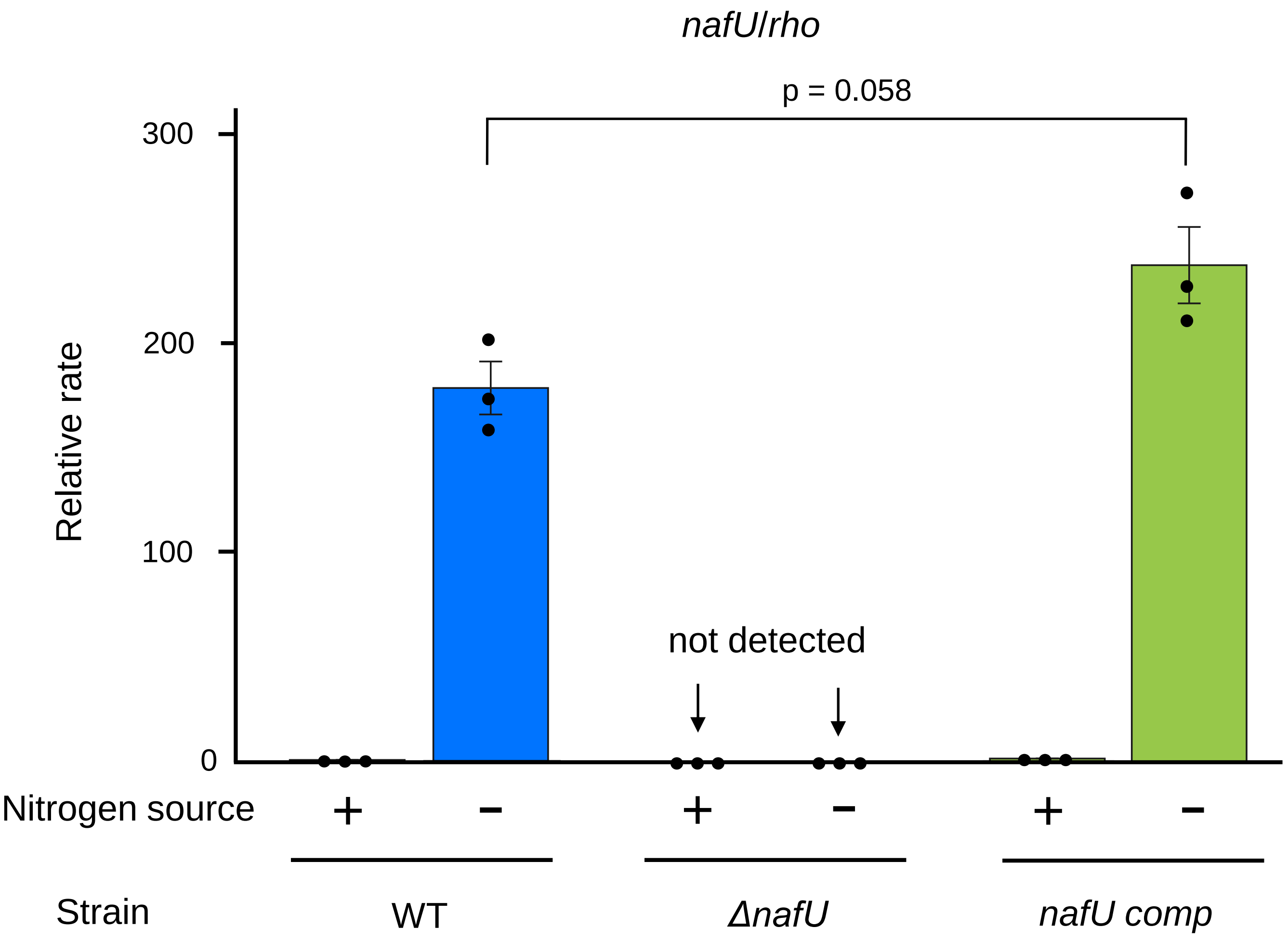

Supplementary Figure 4. The expression levels of *nafU* in each *A. vinelandii* strain 2 h after incubation in MB liquid medium with or without a nitrogen source. The expression levels of *nafU* in wild- type, *ΔnafU*, and *nafU comp* strains were quantified by RT-qPCR. The expression levels of *nafU* in the *ΔnafU* strain were below the range of the calibration curve and could not be quantified (not detected). The expression level of *rho* was used for normalization. Data are shown as relative values when the average expression level of *nafU* in “wild- type strain with nitrogen” is 1. Data are presented as the mean ± SE of three independent experiments. *P-values* were calculated using the Student’s two-sided t-test.

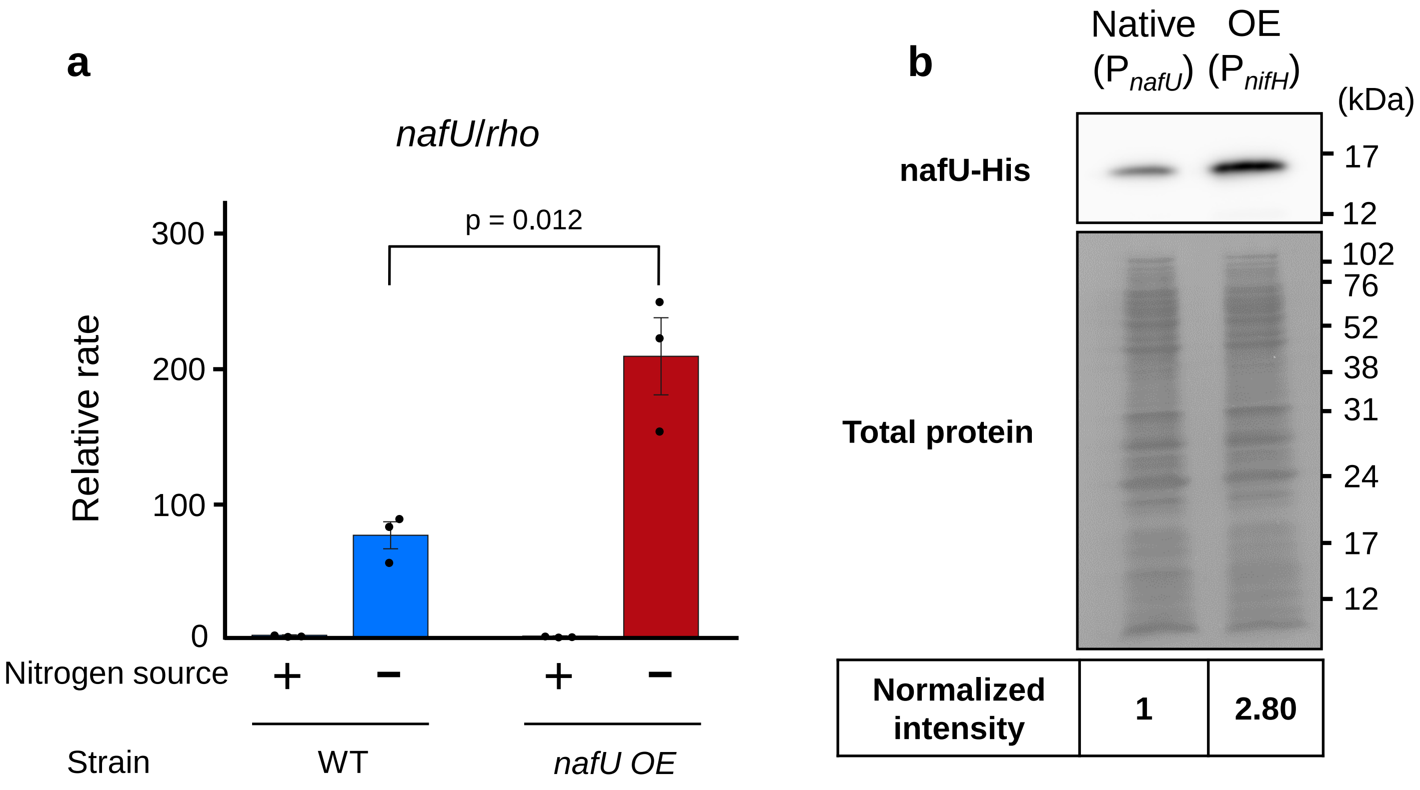

Supplementary Figure 5. The expression levels of *nafU* in WT and *nafU OE* strains. **a** The expression levels of *nafU* in wild-type and *nafU OE* strains at 2 h after the incubation in MB liquid medium were quantified by RT-qPCR. The expression level of *rho* was used for normalization. Data are shown as relative values when the average expression level of *nafU* in “wild- type strain with nitrogen” is 1. Data are presented as the mean ± SE of three independent experiments. *P-values* were calculated using the Student’s two-sided t-test. **b** Western blot quantification of nafU protein in *nafU-His* under native promoter and *nafU OE* strains 4 h after the incubation in MB liquid medium without a nitrogen source. P_{nafU} represents *nafU-His* under native promoter, and P_{nifH} represents *nafU OE*. His-tag was fused to the C-terminus of nafU in both strains to detect the expression using an anti-His-tag antibody. The size of nafU-His tag is 13.8 kDa. Only the membrane proteins were extracted and applied. The quantification of the band intensity in western blot analysis and total protein by CBB staining of the transferred membrane was performed by ImageJ. Before applying, the total protein concentration was measured by BCA assay and the same amount of protein was applied. A full-length blot is presented in Supplementary Figure 12.

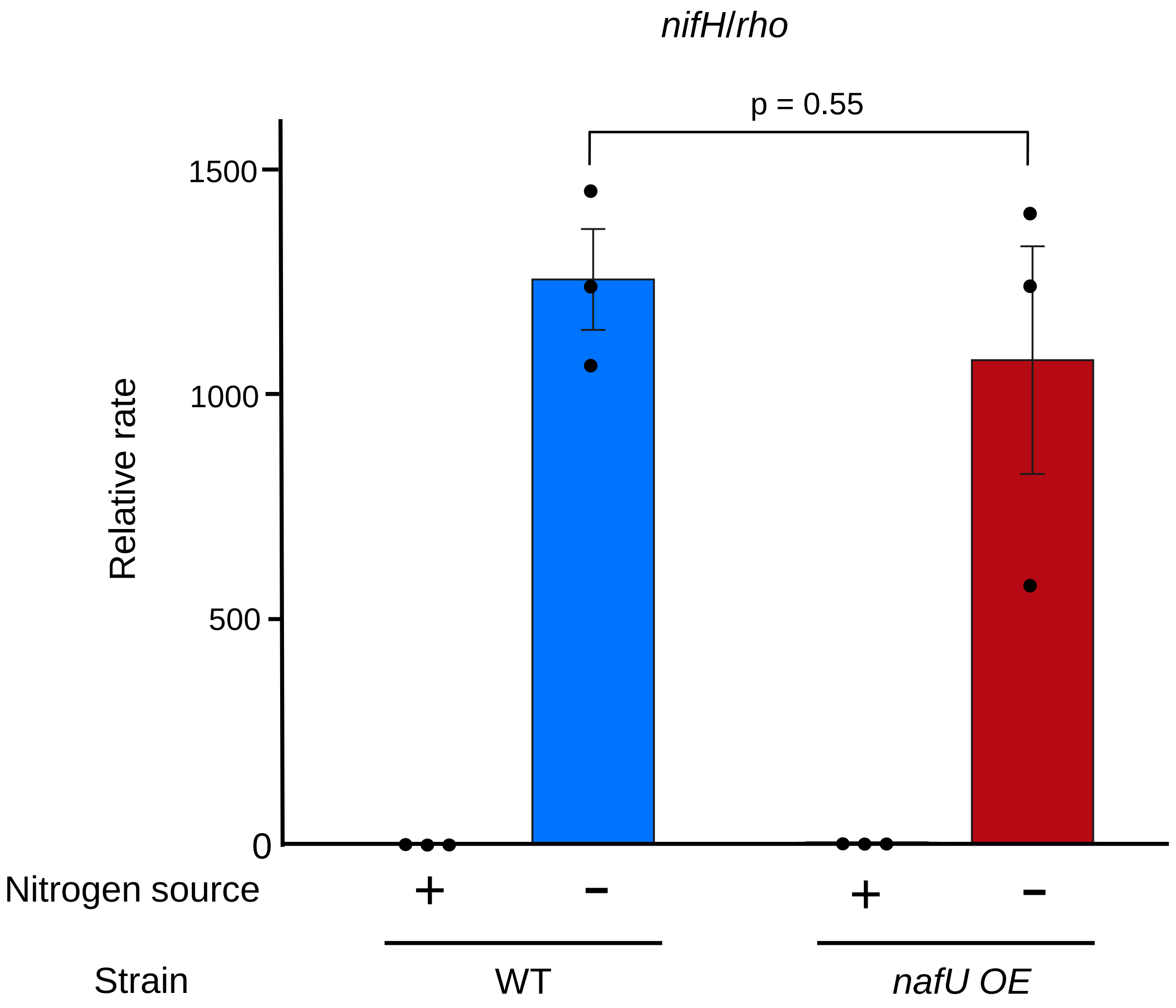

Supplementary Figure 6. Effect of the *nafU* overexpression using *nifH* promoter on the expression of *nifH*. The expression levels of *nifH* in wild-type and *nafU OE* strains at 2 h after the incubation in MB liquid medium with or without a nitrogen source were quantified by RT-qPCR. The expression level of *rho* was used for normalization. Data are shown as relative values when the average expression level of *nifH* in "wild-type strain with nitrogen" is 1. There was no significant difference in the expression level of *nifH* between wild-type and *nafU OE* strains. Data are presented as the mean ± SE of three independent experiments. *P-values* were calculated using the Student's two-sided t-test.

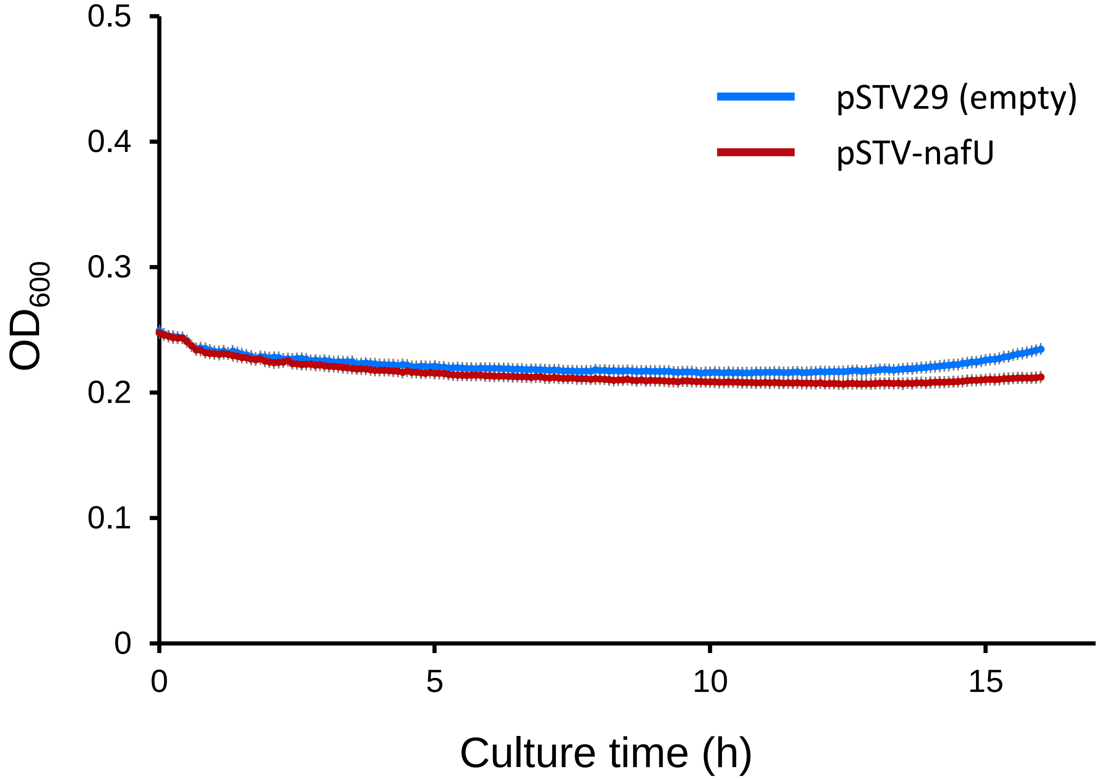

Supplementary Figure 7. The growth curve of nitrogenase-producing *E. coli* (harboring pSTV-nafU) Growth was assessed under the same conditions as the acetylene reduction assay. Data are presented as the mean $\pm$ SE of three independent experiments.

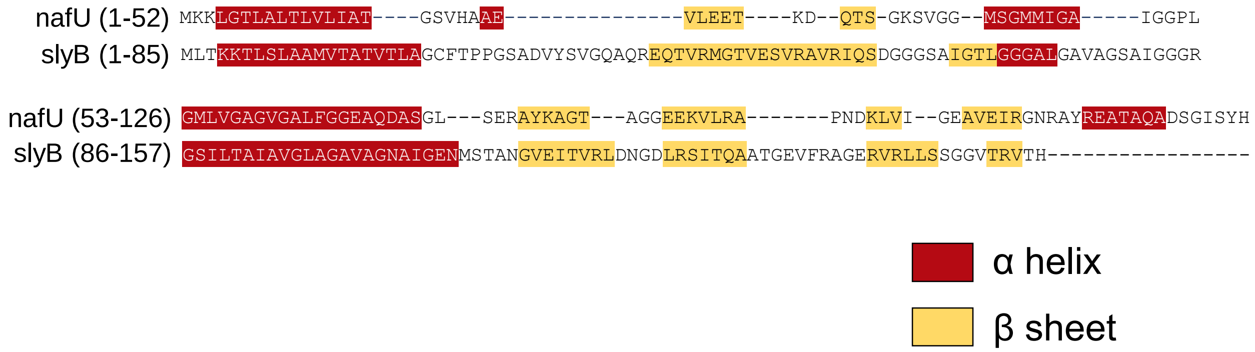

Supplementary Figure 8. The secondary structures of nafU from *A. vinelandii* and slyB from *Burkholderia mutivorans*. Each secondary structure was predicted by JPred4. The alignment was performed based on the predicted secondary structure.

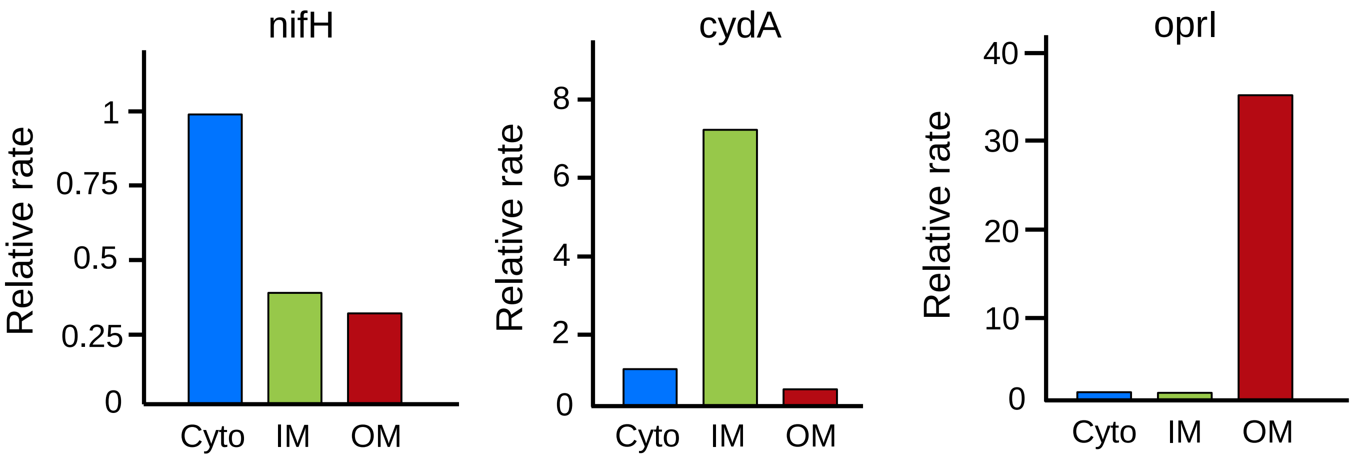

Supplementary Figure 9. Validation of fractionation by quantitative proteome analysis on samples subjected to western blot analysis to confirm the localization of nafU (Figure 4a). The nifH is localized in the cytoplasm, cydA in the inner membrane, and oprI in the outer membrane in *A. vinelandii*. Cyto: cytoplasm fraction, IM: inner membrane fraction, OM: outer membrane fraction. Data are shown as relative values when the value of “cytoplasm fraction” is 1.

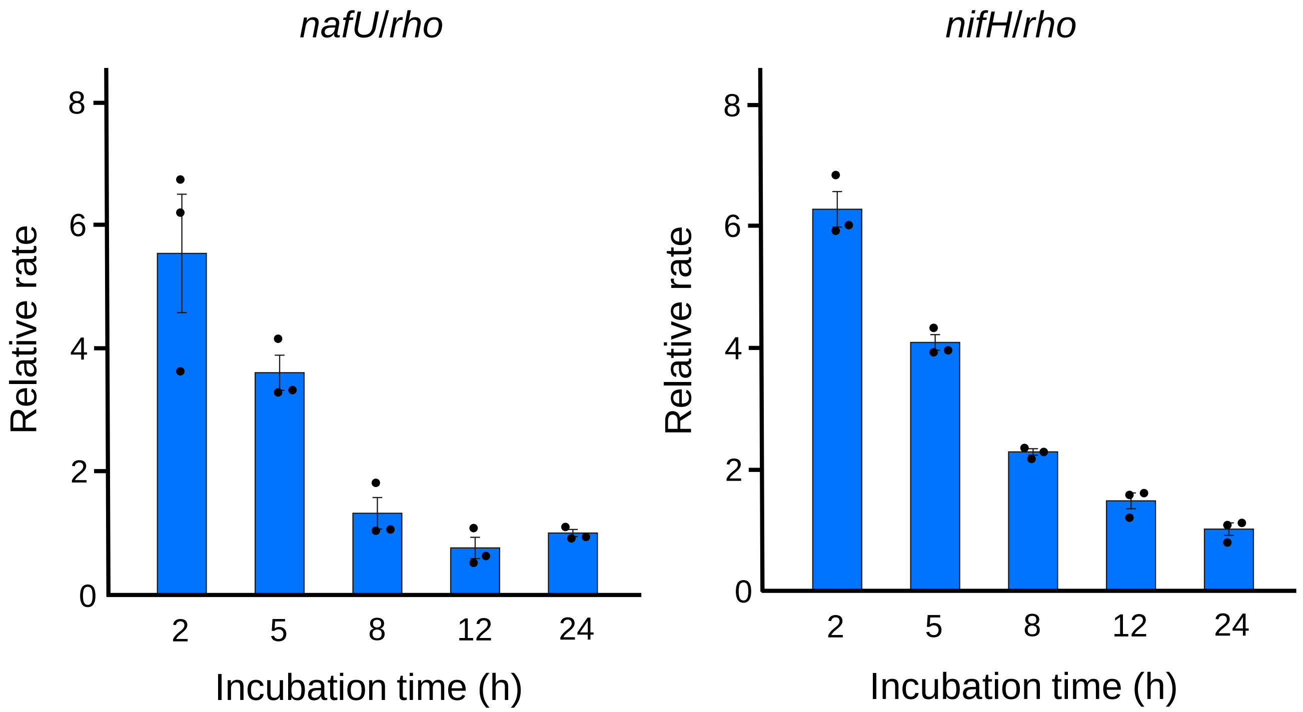

Supplementary Figure 10. Expression changes of *nafU* and *nifH* during the incubation in MB liquid medium without a nitrogen source. The expression levels were quantified by RT-qPCR and the expression level of *rho* was used for normalization. Data are shown as relative values when the expression level of the sample of “24 h after incubation” is 1. Data are presented as the mean ± SE of three independent experiments.

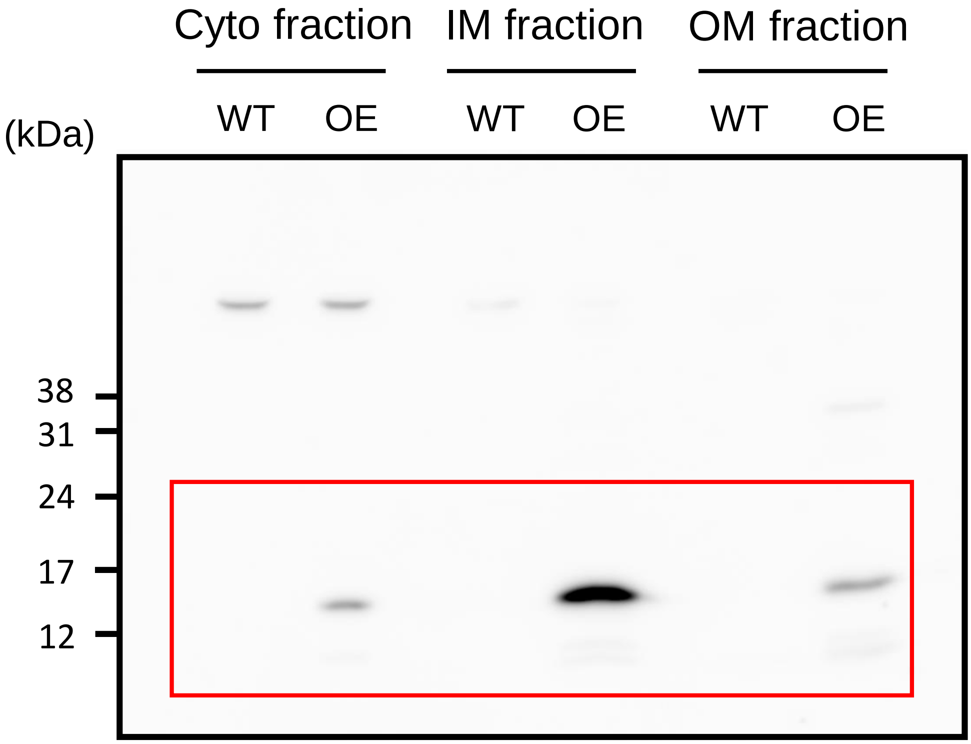

Supplementary Figure 11. Full-length image of western blot analysis (Figure. 4a)

The area surrounded by a red square is the cropped area. Cyto: cytoplasm, IM: inner membrane, OM: outer membrane.

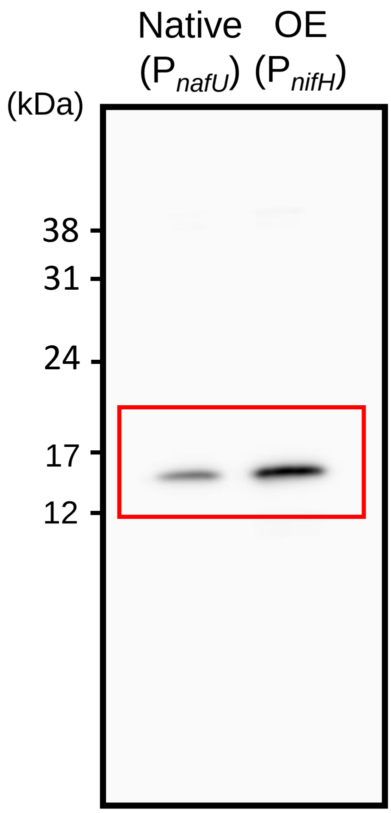

Supplementary Figure 12. Full-length image of western blot analysis (Figure. S5b)

The area surrounded by a red square is the cropped area.
