# Supplementary material for: A critical role of an oxygen-responsive gene for aerobic nitrogenase activity in Azotobacter vinelandii and its application to Escherichia coli
Source: Sci Rep. 2022 Mar 9;12:4182. doi: 10.1038/s41598-022-08007-4 (PMC8907163; doi:10.1038/s41598-022-08007-4)
Supplement: Supplementary file 3 — Supplementary Tables S5 - S6. [file 41598_2022_8007_MOESM3_ESM.pdf]

Table S5. Strains and plasmids used in this study

Strain and plasmid	Genotype or description	Reference
● ***A. vinelandii* strain**		
Azotobacter vinelandii Lipman	Wild type strain	ATCC 9046
ΔnafU	*ΔnafU:: Cm^R*	This study
nafU comp	*ΔnafU* Cm^R ::P_{nafU}-*nafU*-Kan^R	This study
nafU OE	*ΔnafU* Cm^R ::P_{nifH}-*nafU*-His tag-Kan^R	This study
nafU-His under native promoter	*ΔnafU* Cm^R ::P_{nafU}-*nafU*-His tag-Kan^R	This study
● ***E. coli* strain**		
DH5α	F^-, *Φ80dlacZ*ΔM15, Δ(*lacZYA-argF*)U169, *deoR*, *recA*1, *endA*1, *hsdR*17(r_k^-, m_K^+), *phoA*, *supE*44, $λ^-$, *thi*-1, *gyrA*96, *relA*1	Takara Bio
JM109	F' [*traD*36, *proAB*$^+$, *lacI*^{q}, *lacZ*ΔM15] / Δ(*lac-proAB*), *recA*1, *endA*1, *gyrA*96, *thi*-1, *hsdR*17(r_k^-, m_K^+), e14$^-$*(mcrA$^-$), supE*44*, relA*1	Takara Bio
● **plasmid**		
pTrc-nif001	Amp^R, *nifH-nifD-nifK-nifB* under P_{trc} and *iscA-nifU-nifS-nifV-nifQ* under P_{lac}	Tatemichi et al., 2021[39]
pMW-nif002	Kan^R, *nifE-nifN-nifX-nifY* under P_{lac} and *nifW-nifZ-nifM-nifF* under P_{lac}	Tatemichi et al., 2021[39]
pSTV29	Cm^R	Takara Bio
pSTV-nafU	Cm^R, *nafU* under P_{lac}	This study
pUC19-*ΔnafU*	Used for the construction of *ΔnafU*	This study
pUC19-*nafU comp*	Used for the construction of *nafU comp*	This study
pUC19-*nafU OE*	Used for the construction of *nafU OE*	This study
pUC19-*nafU-His* under native promoter	Used for the construction of *nafU-His* under native promoter	This study

Table S6. Primers and the artificial synthesis gene used in this study

- Primers used for RT-qPCR

Primer set		Sequence (5'-3')
nifH	F	CAAGGCCCAGGAAATCTACA
	R	ATACTTCACGATGCCCTTGG
cydA	F	CACGTTCGGGGTGTACTTCT
	R	AACAAGAGCCCGGAGAAAAT
gyrA	F	GGTGGTGCTCAACAACCTCT
	R	AACACCTCGAGCATGTCCTT
nafU	F	TGATAGCTGATGCCGGAGTC
	R	TATTACGCGCACCCAATGAC
rho	F	CGCTTTTCGCATGCTTCTTC
	R	ACCGAACTCAAGCAAAAGCC

- Primers used for the construction of strains

Plasmid name	Primer set		Sequence (5'-3')	Target region or description
pUC19-Δ*nafU*	pUC19	F	GCATGCAAGCTTGGCGTAAT	Linearization of pUC19
		R	GAATTCACTGGCCGTCGTTT	
	nafU DIS A	F	AAACGACGGCCAGTGAATTCAAACGAAAGCTGGGAAGAATTCCTG	647 bp in the downstream of *nafU*
		R	GGCAGTTATTGGTGCCCTTATCACTAAGTGACCCGTTCAAGTCGC	
	nafU DIS B	F	ATCAACAGGGACACCAGGACGAATCCTCCGAGGAAGTCTTCAAT	575 bp in the upstream of *nafU*
		R	ATTACGCCAAGCTTGCATGCAAATGAACTTCACCGAGCACTGTCC	
	Chloramphenicol	F	TAAGGGCACCAATAACTGCC	Chloramphenicol resistance cassette from pSTV29
		R	TCCTGGTGTCCCTGTTGATA	
pUC19-*nafU comp*	pUC19	F	GCATGCAAGCTTGGCGTAAT	Linearization of pUC19
		R	GAATTCACTGGCCGTCGTTT	
	nafU comp A	F	AAACGACGGCCAGTGAATTCAAACGAAAGCTGGGAAGAATTCCTG	The same sequence as *nafU DIS* A
		R	ACCTACACCGAACTGAGATATCACTAAGTGACCCGTTCAAGTCGC	
	nafU comp B	F	AACCTCTTACGTGCCGATCAATATGGTGTCGCTGTGCGGGACAGC	*nafU* + 577 bp in the upstream of *nafU*
		R	ATTACGCCAAGCTTGCATGCAAATGAACTTCACCGAGCACTGTCC	
	Kanamycin	F	TATCTCAGTTCGGTGTAGGTC	Kanamycin resistance cassette from pMW219
		R	ATGATTGAACAAGATGGATTG	
	Cat promoter	F	AATCCATCTTGTTCAATCATTTTAGCTTCCTTAGCTCCTG	*Cat* promoter from pSTV29
		R	TGATCGGCACGTAAGAGGTTCC	

pUC19-*nafU OE*	pUC19	F	GCATGCAAGCTTGGCGTAAT	Linearization of pUC19
		R	GAATTCACTGGCCGTCGTTT	
	nafU OE A	F	AAACGACGGCCAGTGAATTCCGTCTATATCGACGAGGTGGACAAG	1726 bp in the downstream of *nafU*
		R	ACCTACACCGAACTGAGATAGACGGTGAAACAAAGCGCAAGAG	
	nafU OE B	F	CGAATCCTCCGAGGAAGTCTTCAAT	1776 bp in the upstream of *nafU*
		R	ATTACGCCAAGCTTGCATGCGAACGGATGCCGAAAGAAAAACGTG	
	nifH promoter	F	GTGCCGAGCTTTTTCATAGTTAATTTCCTCAGGTTGCATTTCCAC	Promoter of *nifH*
		R	AGACTTCCTCGGAGGATTCGAGGGCAAGAATCGACAACCTATTGC	
	Kan+P_{cat} +terminator+His	F	TATCTCAGTTCGGTGTAGGTCGTTCG	Amplification from pUC19-*nafU comp*
		R	GGTGGTCACCACCACCACCACCACTAAGTGACCCGTTCAAGTCG	
	His+nafU	F	GTGGTGGTGACCACCGGAACCACCGTGATAGCTGATGCCGGAGT	*nafU* + His tag from genome
		R	ATGAAAAAGCTCGGCACCCT	
pUC19-*nafU-His* under native promoter	pUC19	F	GCATGCAAGCTTGGCGTAAT	Linearization of pUC19
		R	GAATTCACTGGCCGTCGTTT	
	nafU Native A	F	AACGACGGCCAGTGAATTCGGCGCAGTGGGGCATCGTCTATATC	1668 bp in the downstream of *nafU*
		R	CCTACACCGAACTGAGATAGCGCGATCCGCAGAGAAACGAAAAG	
	nafU Native B	F	GGTGACCACCGGAACCACCGTGATAGCTGATGCCGGAGTCGGCC	1221 bp in the upstream of *nafU* + *nafU* + His tag
		R	TTACGCCAAGCTTGCATGCACTCCGCCTTGTTCAGGACCAGCAG	
	Kan+P_{cat} +terminator+His	F	TATCTCAGTTCGGTGTAGGTCGTTCG	Amplification from pUC19-*nafU comp*
		R	GGTGGTCACCACCACCACCACCACTAAGTGACCCGTTCAAGTCG	

- Artificial synthesis gene

Gene name	Sequence (5'-3')
nafU gene codon-optimized for *E. coli* (synthesized by IDT)	ATGAAGAAACTTGGAACATTGGCCTTGACACTTGTCTTGATCGCCACTGGTAGCGT<br>ACATGCGGCTGAAGTACTTGAGGAGACTAAGGATCAGACTAGCGGTAAGTCTGTC<br>GGAGGTATGTCCGGGATGATGATCGGGGCGATTGGAGGGCCATTAGGAATGCTG<br>GTGGGAGCGGGGGTAGGGGCGCTTTTTGGTGGCGAAGCGCAAGACGCTTCTGG<br>ATTGAGTGAACGCGCATATAAGGCAGGAACAGCAGGAGGGGAGGAAAAAGTACT<br>GCGCGCACCAAACGACAAGCTGGTAATCGGTGAAGCAGTGGAAATCCGTGGGAA<br>CCGCGCATATCGCGAAGCGACGGCGCAGGCAGACTCGGGCATTTCCTATCATTAA
